# Large‐Scale Forest Restoration Accompanied by Biodiversity Recovery in Costa Rica's Redistributive Payment for Ecosystem Service Program

**DOI:** 10.1111/gcb.70730

**Published:** 2026-02-04

**Authors:** Giacomo L. Delgado, Johan van den Hoogen, Daisy H. Dent, Tom Bradfer‐Lawrence, Leland K. Werden, Rebecca Cole, Cristian Diaz Quesada, Jose‐Angel Jimenez Fajarado, Alberto Méndez Rodríguez, Eduardo Mesén Solorzano, Gilmar Navarrete Chacón, Mario Coto, Irene Suarez Perez, Lucas Vahlas, Yuting Liang, Thomas Ward Crowther

**Affiliations:** ^1^ Institute of Integrative Biology, ETH Zurich Zurich Switzerland; ^2^ Smithsonian Tropical Research Institute Balboa Panama; ^3^ Max Planck Institute of Animal Behavior Konstaz Germany; ^4^ Royal Society for the Protection of Birds Edinburgh UK; ^5^ Costa Rican National Forestry Financing Fund (FONAFIFO) San Jose Costa Rica; ^6^ Costa Rican National System of Protected Areas (SINAC) San Jose Costa Rica; ^7^ Costa Rican National Technical Environmental Secretary (SETENA) San Jose Costa Rica; ^8^ RESTOR Zurich Switzerland; ^9^ Lycée Janson de Sailly, CPGE—BCPST Paris France; ^10^ State Key Laboratory of Soil and Sustainable Agriculture, Institute of Soil Science Chinese Academy of Sciences Nanjing China; ^11^ University of the Chinese Academy of Sciences Beijing China; ^12^ Biodiversity Research and Action for Nature Climate and Humanity (BRANCH) Zug Switzerland; ^13^ King Abdullah University of Science and Technology (KAUST) Thulwal Saudi Arabia

**Keywords:** biodiversity, Costa Rica, ecology, environmental change, forests, inequality, national parks, payments for ecosystem services

## Abstract

Ecosystem restoration is widely recognized as among the most important means to combat both climate change and biodiversity loss. A vast range of studies have illustrated the potential for local ecological recovery, but its efficacy at large spatial scales remains highly uncertain. Until now, the lack of standardized biodiversity monitoring systems has restricted our capacity to evaluate the potential for ecological recovery at large spatial scales. Here, we use an ecoacoustic dataset from 119 locations to evaluate the biodiversity implications of large‐scale forest restoration within Costa Rica's redistributive Payment for Ecosystem Service (PES) program. Soundscapes recorded in degraded pastures were marked by significant changes in the timing and frequencies typical of mature reference forests. In contrast, we show that the acoustic patterns of restored PES sites have recovered and now resemble reference forests more closely. The age and ecological composition of naturally regenerating forests within the PES have led to more dramatic patterns of acoustic recovery (soundscapes 1.4 times more similar to reference forests than pastures) than in their monoculture plantation counterparts (1.24 times). The scale and consistency of these findings provide strong evidence of effective restoration at scale, highlighting the ecological merits of natural restoration approaches over monoculture plantations.

## Graphical abstract

Transforming recorded sound into meaningful ecological insights requires carefully designed methodologies. This workflow diagram describes the steps of our analysis (contained within circles labeled 1–7), which builds on best practices to correct for potential biases. We use this approach to compare soundscape patterns amongst different land use classes and derive insights into the process of ecological recovery. We reveal significant recovery of natural acoustic patterns within the PES, suggesting successful biodiversity restoration has accompanied Costa Rica's success in forest cover restoration.

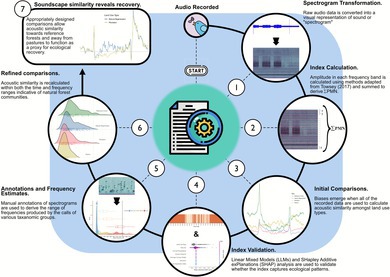

## RESUMEN

La restauración de ecosistemas se reconoce como una de las estrategias más importantes para combatir tanto el cambio climático como la pérdida de biodiversidad. Numerosos estudios han demostrado el potencial de recuperación ecológica a nivel local; sin embargo, su efectividad a gran escala sigue siendo altamente incierta. Hasta ahora, la ausencia de sistemas estandarizados de monitoreo de biodiversidad ha limitado nuestra capacidad para evaluar la recuperación ecológica a gran escala. En este estudio, utilizamos un conjunto de datos ecoacústicos provenientes de 119 sitios para analizar las implicaciones de la restauración forestal sobre la biodiversidad a gran escala, dentro del marco del programa de Pago por Servicios Ambientales (PSA) de Costa Rica. Los paisajes sonoros registrados en pastizales degradados mostraron cambios significativos en los tiempos y frecuencias típicas de los bosques de referencia maduros. En contraste, los sitios restaurados bajo el PSA presentaron patrones acústicos que se asemejan cada vez más a los de los bosques de referencia. Asimismo, la edad y la composición ecológica de los bosques que se regeneran de forma natural dentro del PSA se tradujo en una recuperación acústica más marcada (paisajes sonoros 1,4 veces más similares a los bosques de referencia que a los pastizales) en comparación con las plantaciones de monocultivo (1,24 veces). La consistencia y la magnitud de estos resultados ofrecen evidencia sólida de la eficacia de la restauración a gran escala, al tiempo que destacan los beneficios ecológicos de los enfoques de restauración natural frente a las plantaciones de monocultivo.

## 1 Introduction

Land degradation driven by human extractivism is the largest global driver of biodiversity loss and the source of more than 10% of global carbon emissions (IPBES 2018). The costs and benefits of this extractivism are borne unequally (Boyce 1994; Holland et al. 2009), fueling the overconsumption of the rich (Castano Garcia et al. 2021) while directly threatening the livelihoods of at least 3.2 billion people (IPBES 2018; Vaz et al. 2017). Ecosystem restoration has long been considered a tool to address the ecological crises of biodiversity loss and climate change (Harris et al. 2006) but is now increasingly recognized to have the potential to help address the social crises of inequality and poverty (Andam et al. 2010; Ingram et al. 2014). Recent studies (Chancel et al. 2024; Löfqvist et al. 2023; Miyamoto 2020; Zhen et al. 2014) have gone further to propose that equitably redistributing wealth towards local land stewards is key to achieving effective ecological outcomes. Despite the potential to reverse the drivers of degradation and enable large‐scale ecological recovery, explicitly testing the link between inequality reduction and biodiversity conservation remains challenging. Most notably, we lack empirical evidence of long‐term redistribution paired with tangible environmental outcomes at scale.

Payment for Ecosystem Services (PES) programs, which make direct payments to program participants for ecological improvements, are among the most widely implemented conservation and restoration policies globally (Farley and Costanza 2010). Despite the difficulties in assigning causality to specific policy mechanisms, a large body of literature has associated these programs with reduced deforestation (Jayachandran et al. 2017; Samii et al. 2014; Wiik et al. 2019) and improved human well‐being (Cheng et al. 2023; Ingram et al. 2014; Martin et al. 2014). However, until now, it remains unclear whether these programs can contribute to wide‐scale recovery of ecological intactness (Chen et al. 2020). Assessing the ecological implications of such broad‐scale interventions remains challenging without standardized and ground‐sourced biodiversity indicators. While the cost and complexity of traditional biodiversity surveys are often prohibitive for large‐scale biodiversity assessment (Buxton et al. 2018), the emergence of techniques such as passive acoustic‐monitoring (PAM) has provided a rapid and accessible method to monitor large areas.

**FIGURE 1:**
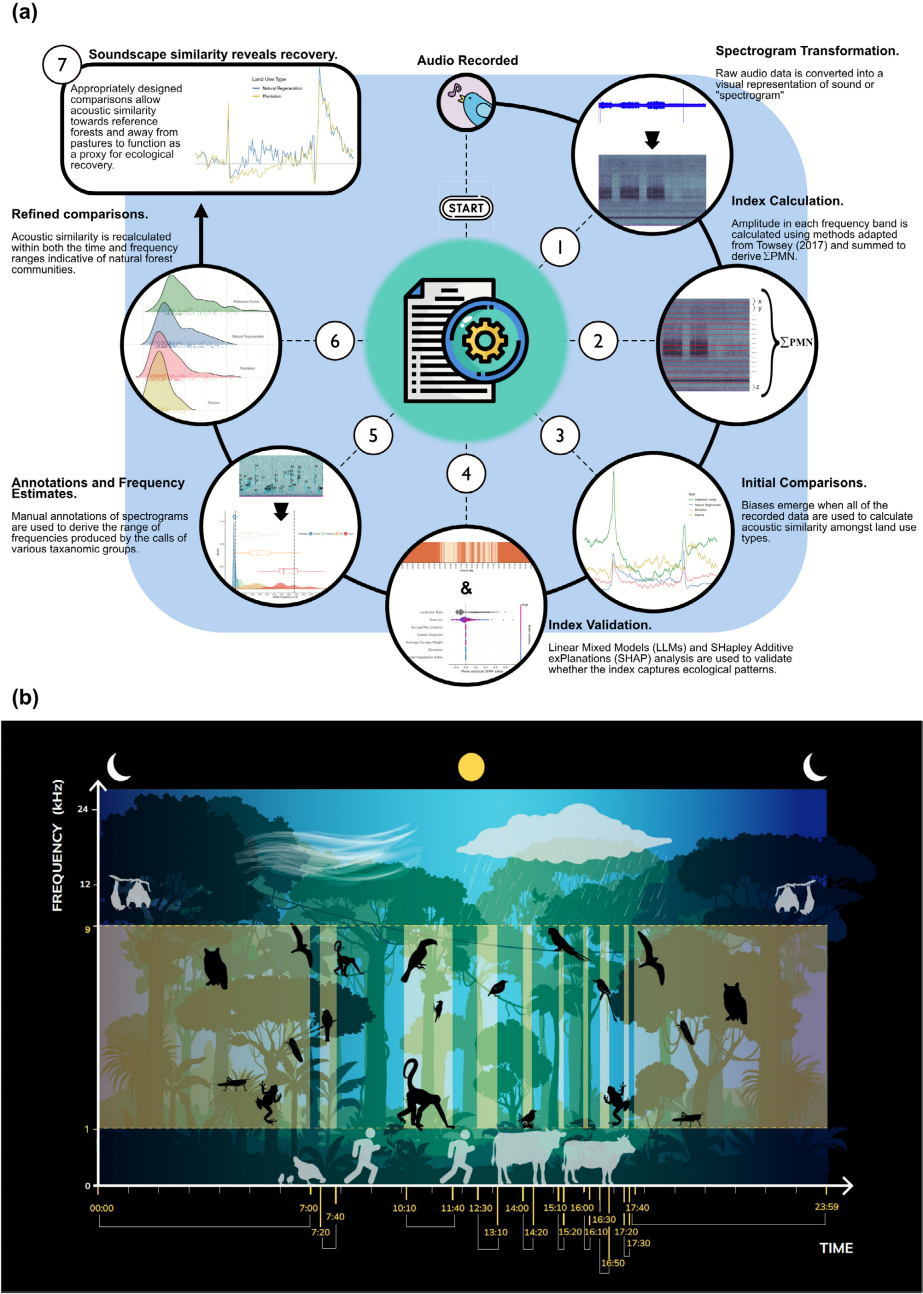
Using soundscape analysis to monitor ecosystem health and biodiversity recovery. (a) Transforming recorded sound into meaningful ecological insights requires carefully designed methodologies. This workflow diagram describes the steps of our analysis, which builds on best practices to correct for potential biases. We use this approach to compare soundscape patterns amongst different land use classes and derive insights into the process of ecological recovery. (b) A main challenge in soundscape analysis stems from the difficulty of dealing with abundant physical and statistical noise. By identifying the times and frequencies associated with biological activity (steps 4 and 5 in (a)), the strength of comparisons can be improved. The natural patterns of the vocalizing fauna in this region of acoustic space (highlighted here in yellow) can be used as a proxy for ecological recovery. Outside of this region of acoustic space, differences between land‐use types are better explained by climatic differences or contain significant acoustic interference.

There exists a rich scientific tradition of using sound in ecological research. Early investigations used sound to understand complex animal behavior like mating, aggression, memory, and learning (Barfield et al. 1979; Beecher and Brenowitz 2005; Searcy et al. 2006). Recent improvements in recording technology and memory storage are allowing ecologists to collect massive datasets “passively” over large spatial and temporal scales (Darras et al. 2025). However, using these datasets requires overcoming acoustic interferences and significant interpretive challenges. Many modern acoustic studies have sought to identify species within recordings in order to monitor populations, track individual animal movements, or estimate species richness (Kelly et al. 2023; Knight et al. 2024; Ribeiro et al. 2022; Wood et al. 2019). However, without exhaustive reference libraries, characterizing each species' sound remains challenging, particularly in highly speciose ecosystems. As such, a large body of studies instead employ soundscape analysis to mathematically summarize entire recordings with the aim of monitoring ecosystem health (Burivalova et al. 2021; Furumo and Mitchell Aide 2019; Müller et al. 2023; Yoh et al. 2024). With ever‐improving analytical practices to strengthen the ecological insights that can be derived from these approaches (Bradfer‐Lawrence et al. 2019, 2023; Jarrett et al. 2025), we now have the capacity to assess how the health of entire ecosystems is changing over time (Figure 1). Yet, until now, no study has employed these methods to evaluate the potential for restoration programs to drive long‐term changes in biodiversity at large spatial scales (i.e., across thousands of km^2^).

Here, we use 16,658 h of ecoacoustic recordings from 119 sites across the entire Nicoya Peninsula (Figure S1) to test whether increases in forest cover have been accompanied by other aspects of ecological recovery in one of the world's most well‐known PES programs: Costa Rica's “Pagamentos por Servicios Ambientales.” We characterize soundscapes of four land use types along a disturbance gradient to assess the ecological recovery of two common PES program interventions. Specifically, the soundscapes of PES sites experiencing “natural regeneration” (native regenerated forests between ~25 and 42 years old) and “timber plantations” (monocultures between ~7 and 20 years old) are assessed, relative to highly disturbed “reference pasture” sites and minimally disturbed “reference forest” sites in protected areas. By assessing the soundscapes of these PES interventions in relation to those of degraded and intact ecosystems, we identify ecological signals of biodiversity recovery within the PES program since its inception 27 years ago. Alongside existing data on forest cover and carbon storage, these results provide the first assessment of the success of this redistributive program in catalyzing large‐scale changes in biodiversity.

## 2 Methods

### 2.1 Study Area

The study was carried out in the Nicoya Peninsula of Costa Rica (Figure S1) between May and July of 2022. The peninsula is the largest in Costa Rica, spanning approximately 51,000 km^2^. It contains both moist and tropical dry forest ecological zones (Holdridge 1967), a pronounced rainy season (May through December), and altitudes ranging from sea level to over 900 m. Livelihoods in the region are largely dependent on the production of beef and participation in a large ecotourism sector, the latter being especially dominant along the Pacific coast.

### 2.2 PES Site Selection

Working with the regional Costa Rican National Forestry Financing Fund (FONAFIFO) office in Nicoya we identified a set of PES contracts appropriate for inclusion in the study. To ensure the independence of acoustic data we set a minimum contract size of 3.5 ha to ensure a distance of 700 m between microphones (see below). We also set a minimum time under contact in the PES of 10 years. FONAFIFO filtered all active contracts in the Nicoya municipal region according to these requirements to create a list of all possible sampling sites, removing any sites deemed inaccessible during the rainy season. Program beneficiaries were contacted and asked for their explicit consent before including their land in the study. We selected sampling sites to ensure that our dataset would be representative of the climatic variation across the Nicoya peninsula.

#### 2.2.1 Natural Regeneration Sites

We sampled 50 sites which FONAFIFO refers to as “Protection of Forests” as they were standing forests at the time of PES application. However, none of these sites included in the study were primary forests, and most, if not all, had regrown on abandoned pastures (Arroyo‐Mora et al. 2005; Calvo‐Alvarado et al. 2009; Sader and Joyce 1988). Additionally, during our informal contact with PES landowners we did not encounter any who had actively restored forests on their farms. Therefore, for consistency and clarity, we refer to these sites as “natural regeneration” as this best describes their ecological identity and management history. While exact forest stand ages were unavailable for these PES sites, previous analyses have found that the region's forests were largely cleared for cattle pasture prior to 1980 and since then have been in a process of dynamic recovery (Arroyo‐Mora et al. 2005; Sader and Joyce 1988). This information suggests that natural regeneration PES sites were between 25 and 42 years at the time of sampling.

#### 2.2.2 Plantation Sites

Of the 51 “Reforestation” sites we sampled, all had been planted with a single species in highly managed timber farms. As such, we refer to these sites as “Plantations.” These monoculture plantations are overwhelmingly planted with *Tectona grandis* and are harvested in 15‐ to 20‐year cycles. The plantations included in this study vary in age from 7 to 20 years old.

### 2.3 Pasture Site Selection

The recent land use history of the Nicoya Peninsula was driven by widespread deforestation for cattle pasture expansion, particularly from 1960 to 1980 (Arroyo‐Mora et al. 2005; Calvo‐Alvarado et al. 2009; Sader and Joyce 1988). The ubiquity of cattle farming in the region has led to pastures that are managed by a diverse set of owners and, consequently, the composition of these pasture ecosystems is also highly variable. They range from diverse mosaics of both remnant trees and naturally occurring grass/shrub species (generally small landowners) to intensively cleared, seasonally flooded, novel grassland habitats planted with exotic grass species (generally larger landowners). We captured this variability in our sampling (Table S1). When possible, we prioritized sampling pastures owned by PES‐participating landowners whose contracts we had also sampled to provide the most appropriate comparisons. The remaining pastures were collected to represent the climatic and geographic variation across the Nicoya Peninsula. We recorded at 20 pasture sites.

### 2.4 Reference Forest Site Selection

To provide a high‐quality baseline for comparison, we sampled 21 mature forests or older secondary forests to represent the ecosystems that would naturally occur in the region in the absence of human interference. We prioritized sampling within protected areas, which have benefited from strict government protection and enforcement. Many of the protected areas sampled overlap with existing estimates of old growth forests in the region (Sader and Joyce 1988), suggesting that they have existed at least since the end of the heavy deforestation period which ended in 1980, if not longer. We estimate our reference forest sites to range from remnant old growth stands to ~42‐year‐old secondary regrowth. We worked with the national park service (SINAC) in the Tempisque conservation area (ACT) to sample protected areas (*n* = 15) and also sampled a handful of private reserves (*n* = 6) in collaboration with the regional FONAFIFO office.

In summary, we sampled 50 naturally regenerating PES sites, 51 plantation PES sites, 20 pastures, and 21 reference forests for a total of 142 sites.

### 2.5 Hardware and Acoustic Recordings

Audiomoth recorders (www.openacousticsdevices.info) versions 1.1 (*n* = 30) and 1.2 (*n* = 20) were used to record at the 142 selected sites. We recorded continuously for six days at each site, with the aim of recording at least 120 h per site (Bradfer‐Lawrence et al. 2019). We used a sample rate of 48 kHz and medium gain, resulting in a captured frequency range of 24 kHz. As such, we were unable to capture bats and other fauna that call in ultrasonic ranges. We wrote files to microSD cards in WAV format.

A single microphone was placed in each PES contract registered with FONAFIFO, regardless of its size. Pastures and reference forests were often larger landscapes of suitable sampling area and when placing multiple recorders within the same continuous area (e.g., in a single national park or within an expanse of pastures), recorders were placed at a minimum distance of 700 m to ensure independence of the data collected. Most distances exceeded 1 km (average distance between sites was 37 km); nevertheless, we retained the 7 site‐pairs with geographic distances below 700 m (Figure S3). Whenever possible, recorders were placed to minimize edge effects and possible interference from rivers, streams, the ocean, human settlements, or high‐traffic motorways. However, this was not always possible, and some recordings were excluded due to high levels of acoustic interference.

Recorders were attached to trees at approximately 2 m height. The lack of woody vegetation in pastures required us to occasionally attach recorders to fence posts and at lower heights. Forest canopy height was then taken using a laser‐range finder three times along a 10 m transect centered around the recorder. The first and last 30 min recorded by each audiomoth were excluded from analysis to avoid capturing sounds created during fieldwork.

Hardware failure or excessive acoustic interference resulted in incomplete, low‐quality, or failed recordings at 23 sites, such that all further analyses include a total of 119 sites: 18 Reference Forest, 39 Natural Regeneration, 43 Plantation, and 19 Pasture sites. Recorder behavior also resulted in few data points for 3 min of the day (minutes 385, 386, and 1080), so we excluded these minutes from all analyses.

### 2.6 Acoustic Processing and Index Calculation

We collected a total of 999,470 unique minutes, or nearly 2 years, of audio data. Using R (version 4.1.2), the seewave (Sueur et al. 2008) and soundecology packages, we calculated a modified version of the Power Minus Noise (PMNsp) statistic as described in Towsey (2017). PMNsp is a vector derived from a spectrogram from which the modal amplitude (or energy) per frequency band (*n* = 256 per recording, assuming a FFT of 512) is removed (this modal value represents the background noise or BGNsp, which we retain as a predictor in our linear mixed models [see below]). The de‐noised spectrogram is then smoothed (see Towsey 2017 for details), and the resulting maximum amplitude value per band is retained as PMNsp for every minute. While the BGNsp removal eliminates constant geophonies (e.g., rain and wind), it also removes some constant insect‐produced noises (e.g., cicada calls), but more variable insect noises are retained. Rather than the difference between the maximum decibel value and BGNsp value for each frequency band (as in PMNsp), we took the sum of differences between amplitude values and the BGNsp across all time bins (*n* = 5625, each ~0.01 s), for each band and minute to derive ΣPMN.

When aggregating the index across frequencies, ΣPMN values for each 93.75 Hz band were summed to obtain values for larger frequency ranges (e.g., summing all 256 bands gives a measure of acoustic energy across the entire frequency spectrum recorded). When aggregating the index across matching minutes (see below), values were averaged to produce a mean value for said minute. Within sites this was done across the approximately six days of recording to derive an average diel soundscape. For comparisons between sites, ΣPMN was often further averaged into 10‐min bins within each site (see below). Thus, our modified index was used to provide a descriptive measure of total acoustic power for the intersection of a 1 or 10‐min bin and any frequency range of interest.

### 2.7 Index Visualization and Predictive Variable Modeling

**FIGURE 2:**
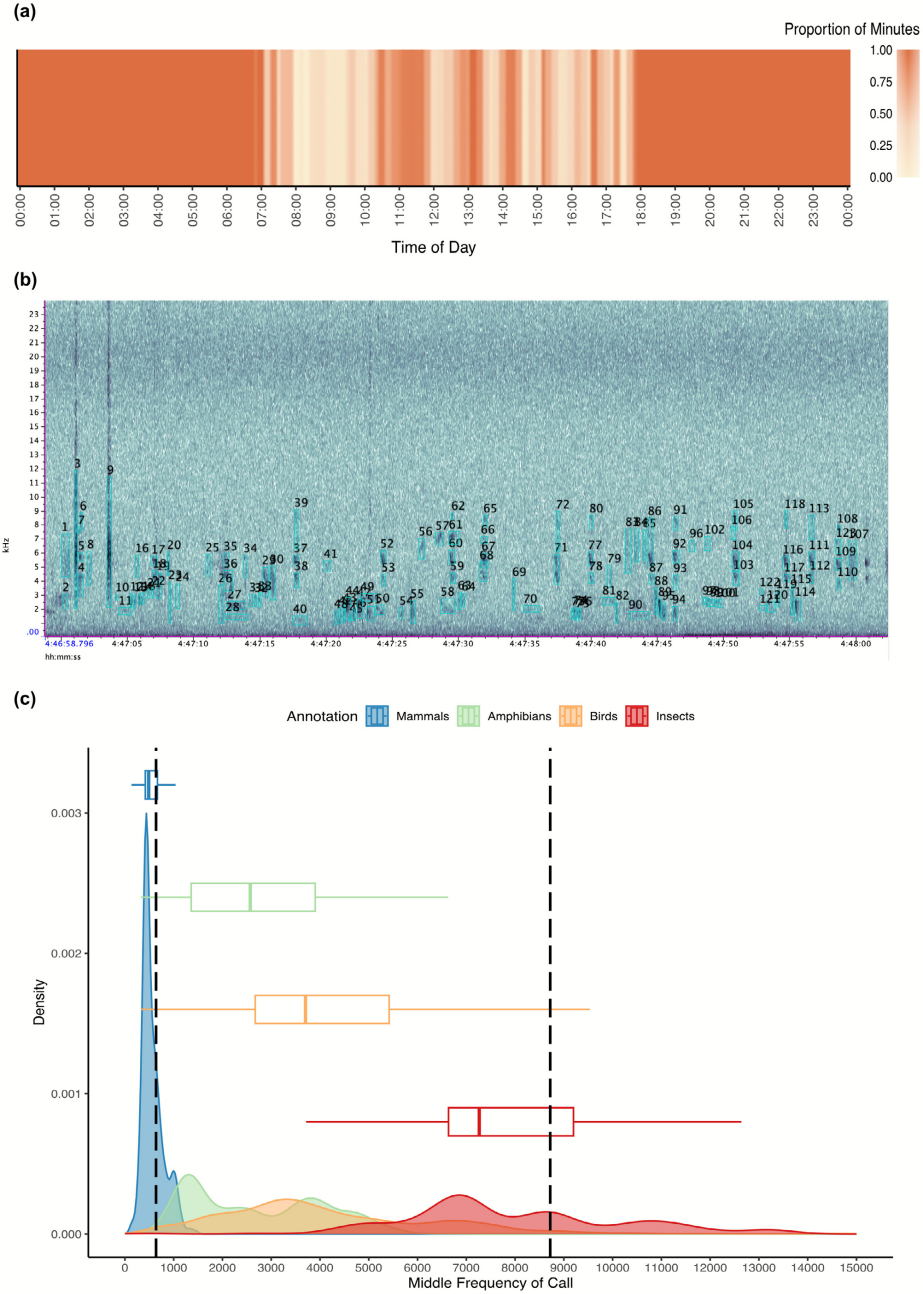
Improving the performance of acoustic indices requires applying them contextually to relevant areas of acoustic space. (a) Models were applied separately to every minute of the day to determine when meaningful comparisons can be made. Minutes that contain land‐use type as an important predictive variable are shown here, grouped into 10‐min bins, and colored by proportion. Time bins where at least 50% of individual minute models contained land‐use type as a predictor were used in subsequent analysis. (b) To identify the ecologically relevant frequencies we annotated 120 min of data in the software RAVEN, such that we could estimate the frequency ranges of taxonomic groups of interest. One example minute of these annotations is shown. Every individual sound element is selected manually (light blue boxes). (c) These annotations are then used to derive frequency ranges for four taxonomic classes. The densities (shaded curves) and average (boxplots) calls for each class are plotted. The overall biophonic range is marked by vertical black dotted lines.

To test the impacts of a set of predictive variables on ΣPMN we used two approaches (Figure 2). First, we used gradient boosted machines (Friedman 2002) to evaluate whether the index was capturing ecological attributes driven by the land‐use history of land use types (Figure S2). This approach allowed us to quantify the relative importance of each of 7 predictive variables on predicted ΣPMN: land‐use type, time (10‐min bins), annual precipitation, enhanced vegetation index (EVI), human modification, elevation, and mean canopy height. We aggregated ΣPMN to obtain a single value for every 10 min (e.g., 5:00–5:10 a.m.) for all recording days for every site. The variable importance of each predictive variable was then assessed via Shapley additive explanations (SHAP) analysis (Lundberg and Lee 2017) (Figure S2). To avoid overestimating the importance of our categorical variable for land use, we included each land‐use type as a binary variable (e.g., reference forest: TRUE/FALSE) and aggregated the SHAP importance of the four classes into a single variable without direction.

Second, we adapted methods from Burivalova et al. (2019) to identify the times during which acoustic patterns were driven primarily by these ecological differences using linear mixed models. We constructed linear mixed models using per‐minute ΣPMN values. Every minute of data was individually fitted with 128 candidate models, one for every possible combination of 7 predictive variables (same as those mentioned above but replacing time with BGNsp, which was aggregated as described above for ΣPMN). For each minute, we selected the single model with the lowest Akaike Information Criterion (AIC) score (Aikake i.e., the most parsimonious fit). Hardware identity (v1.1 vs. v1.2 audiomoth recorder) was also included in every model as a random factor to account for potential technological differences (Jarrett et al. 2025). Using this approach allowed us to eliminate minutes in which ecological differences among land use types were obscured by literal and statistical noise. Any minute in which “land‐use type” was not retained in the final model was omitted from subsequent analysis (Figure 2a). In any subsequent steps of the analysis in which ΣPMN was aggregated into 10‐min time bins, we included any 10‐min bin in which more than half of minute‐specific models contained site type as a predictor (Figure 2a).

### 2.8 Taxonomic Group Frequency Range Estimations

We derived dataset‐specific estimates of the frequency ranges occupied by several taxonomic groups by manually annotating 120 min of recordings (Figure 2b). To avoid biased estimations resulting from species with temporally restricted calling behavior, a random number generator was used to pick minutes and sites representing the full 24‐h cycle (60 diurnal and 60 nocturnal) and land‐use types (30 for each). Chosen minutes were loaded into Raven (Cornell Ornithology Lab) where every visible and audible sonotype, or individual sound element, was carefully selected to measure the maximum and minimum frequencies and assigned to one of five taxonomic groups (Humans/Domestic Animals, Mammals, Amphibians, Birds, or Insects; Figure 2c). Anthropophony (sounds of human origin) and calls from domestic animals made up the majority (68%) of sounds in the 0–1 kHz range. The significant overlap with wild mammals in these frequencies limits the interpretability of results for this low range, and we excluded this frequency band from further analyses.

Any sounds that could not be identified with certainty by the authorship team were marked as “unknown.” The resulting frequency ranges for each taxonomic group were then exported into R where the mean frequency characteristics (mean minimum, maximum and midpoint frequency) and occupancy densities per taxonomic group were calculated (Figure S3b). We used an ANOVA and post hoc pairwise test to determine whether mean frequency characteristics differed significantly between taxonomic groups.

### 2.9 Acoustic Similarity

**FIGURE 3:**
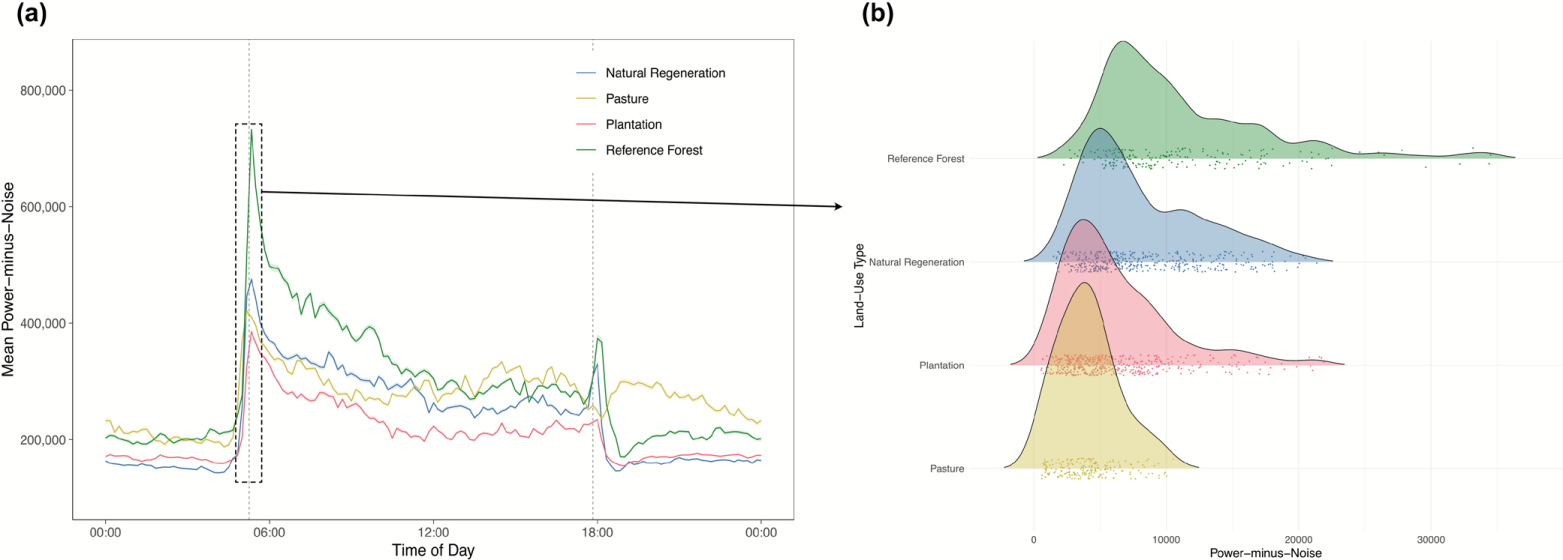
Comparing soundscapes among land‐use types. (a) Average ΣPMN patterns aggregated for the frequency range 1–9 kHz are plotted for each land use type. Shaded colors around lines indicate standard errors of the means ($\mathrm{SEM}=\frac{\mathrm{SD}}{\surd N}$). However, meaningful acoustic comparisons can only be made at matching time‐frequency intersections. (b) We derive measures of acoustic similarity by comparing the distributions (each colored point represents a minute of data from a single site) of all land‐use types (colored curves), in each of 880 time‐frequency intersections. The Wasserstein or earth‐mover's distance calculates the magnitude and distance require to move one distribution into another. A single set of distributions, colored by land‐use type, are shown for the 3–4 kHz and 5:15–5:25 a.m. intersection (i.e., the most acoustically active frequency band during the peak of the dawn chorus).

To assess the similarity of treatment and reference sites, we analyzed the soundscapes including all recorded frequencies, during only the dawn chorus (hours between 5:00 and 6:30 a.m.) and then again after identifying the most ecologically relevant regions of acoustic space (Figure 1a). These comparisons provide insights into the robustness of the observed patterns and the importance of applying acoustic indices at ecologically meaningful times and frequencies (Metcalf et al. 2021). We calculated acoustic similarity (derived from Wasserstein distances) among land‐use types between each matching set of time‐frequency intersections (for the focal region of acoustic space this amounted to 880 comparisons, 110 time bins × 8 one kHz frequency bands between 1 and 9 kHz). Distance metrics were calculated from every land‐use type to both baseline ecosystems (Figure 3). We used a Friedman non‐parametric statistical test for repeated measures to detect differences in distances across 10‐min time bins and a Wilcoxon signed‐rank post hoc test with Bonferroni correction to identify significant differences among land‐use types.

**FIGURE 4:**
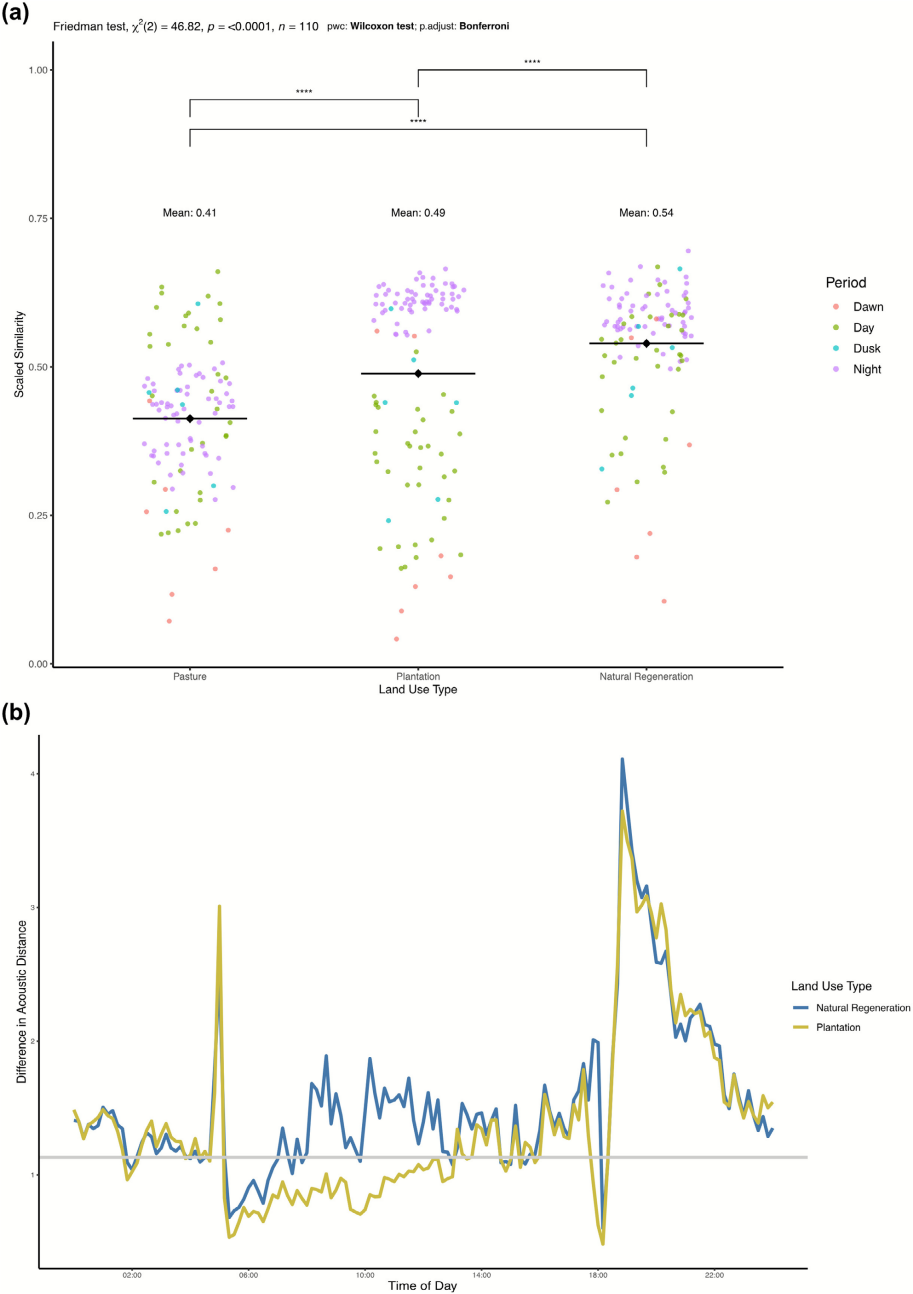
Soundscape similarity reveals substantial recovery in PES natural regeneration sites. Within the identified 880 time‐frequency intersections, high similarity to reference forests can function as a proxy for ecological recovery. (a) Overall similarities to reference forests are plotted for the other three land use types. Higher values indicate more shared acoustic characteristics. Each point represents the mean similarity in a single time bin, colored by the period of the day. Black horizontal lines indicate land‐use means and brackets indicate the results of a Wilcoxon post hoc statistical test. (b) However, overall similarity values average out important patterns within matching comparisons. Within each time‐matched comparison we plot how much more acoustically similar natural regeneration sites (blue) and plantations (yellow) are to reference forests or pastures ($\frac{\text{Similarity Reference Forest}}{\text{Similarity Pastures}}$). Values equal to 1 imply equivalent acoustic distances to reference forests and pastures, values greater than 1 indicate a skew towards reference forests and values below 1 indicate a skew towards pastures. Here we plot these comparisons for the entire diel cycle, although results are reported only for the 110 identified time bins.

These distances are reported as similarity values after having applied an inverse exponential scaling of $y=e^{-\mathrm{kx}}$, where *x* is the calculated distance and *k* = 0.00001 (Figure 4a). Values of 1 indicate complete overlap and values close to 0 indicate large acoustic dissimilarity. Additionally, for every matching time‐frequency intersection, we calculated the relative skew in distance towards reference forests and away from pastures (Figure 4b). We then took the mean of these percentages across all time‐frequency intersections to evaluate how much closer PES classes were to one baseline than another.

## 3 Results

### 3.1 Utility of Power‐Minus‐Noise Index; ΣPMN

Using the Friedman test for repeated measures, we detected significant soundscape differences among the average daily ΣPMN patterns for all land‐use types (*x* ^2^ = 320; *p* < 0.001). A SHAP analysis conducted on the resulting model ranked time and land‐use type as the most impactful variables for predicting ΣPMN. SHAP values for these two variables were multiple orders of magnitude larger than those of any other tested variable (Figure S2).

### 3.2 Initial Acoustic Comparisons

Initial acoustic comparisons using all the acoustic data we collected suggested substantial recovery in PES sites. Soundscape similarity to reference forests was significantly larger (0.537 and 0.455) than similarity to pastures (0.435 and 0.415) for both natural regeneration sites and plantation sites, respectively (Table S1). Restricting this same analysis to only the hours between 5:00 and 6:30 a.m. (i.e., the dawn chorus) resulted in both natural regeneration sites and plantations exhibiting significantly larger similarities to pastures (0.373 and 0.335 respectively) than to reference forests (0.298 and 0.184; Table S2). Significant changes in the results were also observed when making comparisons using the full recorded range of acoustic frequencies (Table S3).

### 3.3 Identification of Biologically Relevant Acoustic Space

In 77% of minutes, the linear mixed models selected using the Akaike information criterion (AIC) (Akaike 1987) included land‐use as an important predictive variable (Figure 2a). Specifically, these include all nocturnal, dusk and dawn hours (i.e., 30 min before and after sunrise and sunset respectively) between 17:20 and 7:40 (excluding two 10‐min bins) and various smaller ranges during the day (10:10–11:40, 12:30–13:10, 14:00–14:20, 15:10–15:20, 16:00–16:10 and 16:30–16:50) (Figure 1b), for a total of 110 time bins.

Our manual annotations (Figure 2b,c) resulted in 8543 sound elements (sonotypes) identified to the taxon level. As predicted by the Acoustic Niche Hypothesis (Krause 1993), there was significant frequency partitioning between the four taxonomic groups of interest (ANOVA; *F* = 1437, *p* < 0.01), with 90% of calls for each taxa in the following ranges: mammals 318 and 1036 Hz, amphibians 1132 and 4895 Hz, birds 1261 and 8004 Hz, and insects 4831 and 12,321 Hz (Figure 2c). Of the sonotypes annotated, 90% of all biophony (i.e., biological sound) fell within a range of 638 and 8721 Hz. Thus, we considered 0–9 kHz to be the biologically active range within our dataset, an estimate that is consistent with previous research (Boelman et al. 2007). Acoustic patterns within and between land use classes were highly frequency specific (Figure S4).

### 3.4 Soundscape Similarity

Within the identified region of acoustic space, soundscape patterns between pastures (i.e., most disturbed) and mature forests (i.e., minimally disturbed) were highly dissimilar (mean similarity 0.413; Figure 4a). Temporally, differences between pastures and reference forests were most pronounced in the time‐bins during the dawn (mean similarity of 0.224, minimum of 0.07) and the night (mean of 0.415), alongside low similarity values in the 5–6 and 7–8 kHz bands. Notably, pastures exhibit a flattened dusk peak that occurred later and lasted longer (Figure 3a) compared to other land use types, rather than the typical strong and sharp peak in dusk acoustic activity. This led to low similarity values between pastures and reference forests stretching into the early night.

Among the PES sites, natural regeneration sites shared considerable acoustic characteristics with reference forests (mean similarity of 0.539; Figure 4a). This was significantly larger (*p* < 0.01; Table S4) than the similarities between either reference forests and pastures (mean similarity 0.413) or reference forest and plantations (mean of 0.489). The similarities peaked during the early afternoon and particularly during the dusk chorus, where mean similarity values regularly exceeded 0.90, indicating almost complete acoustic overlap. These similarities were strongest in the mid and high frequency bands which are most densely occupied by vocalizing birds, amphibians and insects (3–4, 6–7 kHz; Figure S4). Overall, for any given time‐matched comparison, naturally regenerating forests were on average 1.4 times, and as much as 4 times, more similar to reference forests than pastures (Figure 4b). This skew towards reference forests was found in 80% of time bins (88 of 110). The only exception to this pattern was in the dawn chorus, during which natural regeneration sites were more similar to pastures than to reference forests (Figure 4b). Overall natural regeneration sites exhibited a mean similarity to pastures of 0.429, which was significantly smaller (*p* < 0.01) than the mean similarity to reference forests (0.54).

Plantation sites within the PES exhibited intermediate similarities of 0.489 and 0.42 to reference forests and pastures respectively. In comparison to natural regeneration sites, plantations were significantly less similar (*p* < 0.01) to reference forests (Figure 4a). The acoustic patterns captured in plantations are characterized by consistently reduced levels of biological activity across the entire diel cycle (Figure S4), indicating an overall lack of vocalizing biodiversity. Plantations skewed towards reference forests in 64.5% of time bins (compared to the 80% for natural regeneration sites) and were on average 1.24 times closer to reference forests than pastures at any given time (Figure 4b). These similarities to reference forests were highly variable. We found plantations to have high nocturnal and low dawn similarity values, consistent with the patterns observed in natural regeneration sites. However, unlike natural regeneration sites, plantations skewed heavily towards pastures during the entire diurnal period (Figure 4b), perhaps because of their shared identity as heavily managed and human occupied ecosystems. Even so, plantations and natural regeneration sites did not significantly differ in their mean similarity to pastures.

## 4 Discussion

Despite international recognition of the need to halt and reverse the loss of biodiversity, empirical evidence of successful broadscale restoration of terrestrial biodiversity remains scarce (Brancalion et al. 2025; Romanelli et al. 2022; Rozendaal et al. 2019). We used acoustic data to identify the ecological signals of ecosystem recovery in Costa Rica's Payment for Ecosystem Services program across a large geographic extent (Figure S1). We found that the redistributive implementation of Costa Rica's PES program successfully moved the soundscapes of naturally regenerating forests closer to reference mature forests and away from pre‐intervention pastures (Figure 4). Specifically, natural regenerating forests in the program were on average 1.4 times closer to reference forests than they were to pastures (Figure 4b). These results provide tangible evidence of ecological recovery at a broad scale, suggest biodiversity uplift within the PES program, and provide empirical support for equitable wealth redistribution as an effective financing mechanism for restoration under certain contexts.

### 4.1 Soundscapes Are Habitat Specific

To track the biodiversity impacts of Costa Rica's national‐scale restoration program, we analyzed the unique acoustic signatures, or soundscapes, of sites across a recovery gradient. Soundscapes are rich in both ecological (Pijanowski et al. 2011; Sueur and Farina 2015) and functional (Krause 1993; Marten and Marler 1977) information, but the interpretation of acoustic information remains difficult (Bradfer‐Lawrence, Buřivalová, and Dent 2025). Past studies have often relied on the use of acoustic indices to simplify the complexity contained within increasingly massive acoustic datasets (Bradfer‐Lawrence, Duthie, et al. 2025). Many acoustic indices exist, but their complexity often makes them highly context‐dependent and difficult to interpret, potentially leading to misunderstanding and misapplication (Alcocer et al. 2022; Bradfer‐Lawrence, Buřivalová, and Dent 2025; Buxton et al. 2018). As such, the utility of these indices to describe patterns of biodiversity remains highly contested (Alcocer et al. 2022; Sethi et al. 2023), notably because their associations with measures of species richness are weak. Therefore, rather than attempting to translate soundscape complexity directly into biological diversity per se, we derive a modification of the “Power Minus Noise” statistic (PMNsp) (Towsey 2017), which describes a soundscape's energy or “loudness.”

The interpretation of this index is straightforward, with high values indicating soundscapes with high, complex, and variable energy levels, such that individual sounds regularly rise above the modal amplitude value. As expected, recordings during the dawn chorus exhibited the highest values within our dataset. Low values of ΣPMN indicate modal and maximum amplitude values that approach each other, either because both remain low (i.e., silence) or because the soundscape is completely saturated with high levels of acoustic energy (i.e., BGNsp is high). The latter case often results from noises that are constant across the entire recording (e.g., cicada calls and rainstorms) such that despite extremely loud recordings, ΣPMN remains low. Thus, ΣPMN is readily tied to both observed soundscape patterns and underlying biological events. Indeed, we confirmed the utility of our index for capturing both daily fluctuations in acoustic energy (Figure S2) and the long‐term differences in acoustic patterns driven by changes in ecological condition (Figure 2). Ecological inferences can then be made by comparing between soundscapes at matching time‐frequency intersections.

### 4.2 Analytical Specificity Strengthens Ecological Inferences

Having established the utility of our index, we aimed to quantify the levels of acoustic recovery within PES sites. Due to the temporal and spectral specificity of acoustic patterns, meaningful soundscape comparisons can only be made at matching times and frequencies (Figure 1). To illustrate the importance of these analytical considerations, we began by conducting comparisons using all the acoustic data we collected (i.e., likely to include significant literal and statistical noise). The results suggest exaggerated levels of recovery in PES sites (Table S1). However, restricting this same analysis to only the dawn chorus (a practice not uncommon in previous acoustic studies), the opposite trend emerges, with PES sites resembling pastures more than they do reference forests, suggesting little to any recovery has taken place (Table S2). These apparently contradictory results emphasize the importance of applying acoustic indices contextually to the most biologically relevant areas of the acoustic space (i.e., time and frequency; Figure 1). Comparisons made using all recorded data indiscriminately may serve to “average” out important differences, while heavily time‐restricted comparisons may not capture the uneven nature of ecological recovery.

When appropriate comparisons among soundscapes are made, the resulting patterns have been shown to underlie differences in species assemblages and beta diversity (Burivalova et al. 2019; Müller et al. 2023). Employing this analytical specificity allows acoustic similarity to reference forests (minimally disturbed/post‐restoration state) and away from pastures (heavily disturbed/pre‐restoration state) to function as a proxy for ecological recovery (Figure 4). We reiterate past research that recommends acoustic studies explicitly account for multiple aspects of soundscape variation (e.g., time, frequency, amplitude) to improve ecological conclusions (Bradfer‐Lawrence, Duthie, et al. 2025; Metcalf et al. 2021; Figure 2).

To improve our own comparisons, we empirically identify a focal region of acoustic space (Figure 1b), within which, evidence suggests acoustic similarity may correlate with patterns of ecological diversity and recovery (Znidersic and Watson 2022). First, we derive the frequency ranges occupied by biological sound. While other studies have used a priori knowledge or literature‐based estimates (Metcalf et al. 2021), we manually annotated over 8000 individual sound elements to reveal patterns that are consistent with acoustic niches (Krause 1993), occupied by distinct taxonomic classes. Specifically, mammals and amphibians largely call in the lower frequency bands, while birds and insects have highly diverse calls that extend into higher frequency ranges (Figure 2c). However, we found humans disturb these natural acoustic dynamics by directly occupying acoustic niches (i.e., noise pollution) in lower frequencies and perhaps indirectly by disturbing ecosystems, leading to altered acoustic dynamics among land‐use types. Adapting methods from Burivalova et al. (2019), we also identified the times of day where ecological variables, rather than climactic ones, drove soundscape patterns. The times identified largely fell during the evening and early morning hours and suggest that acoustic patterns outside of these times may be driven by human acoustic interference in the mid‐morning and the typical mid‐afternoon rain (Figure 2a). We calculated soundscape similarity only within the time‐frequency intersections of this identified region of acoustic space.

### 4.3 Comparing Soundscapes to Track Ecological Recovery

Focusing on both the time and frequency ranges indicative of natural forest communities (Figure 1), we compared soundscapes recorded from four distinct land‐use types (Figure 3). Reference forest soundscapes were rich in spectral diversity (i.e., acoustic energy distributed across many frequency ranges; Figure S4) and had predictable temporal patterns with strong peaks in energy during early morning and evening hours associated with dawn and dusk choruses. In contrast to reference forests, pastures were marked by significant human influence and altered soundscape dynamics, especially temporally, with a loss of dawn and dusk choruses and louder nocturnal soundscapes (Figure 3a). Such loss of acoustic temporal structures has been previously associated with heavily disturbed ecosystems (Burivalova et al. 2022; Marín‐Gómez et al. 2020; Pease and Gilbert 2025). Additionally, our measure of acoustic similarity revealed low similarity values across all times in the 5–6 and 7–8 kHz bands, consistent with findings that heavily disturbed tropical pastures exhibit lower diversity and altered composition of bird (MacGregor‐Fors and Schondube 2011; Maya‐Elizarrarás and Schondube 2015) and insect (Brown 1997; Paiva et al. 2020) communities. Taken together, the acoustic changes observed in pastures suggest modified community assemblages. In addition to the loss of native faunal abundance and/or diversity, it may also be possible that the novel ecosystems these seasonally flooded grasslands provide attract specific taxa (e.g., anurans) whose vocalizations change acoustic dynamics (Pulsford et al. 2019).

We found that the soundscapes of natural regeneration sites in the PES program were, at any given time and frequency, significantly closer to reference forests than they were to the pastures from which they recovered, up to 4 times as much (Figure 4b). This mirrors findings for other tropical secondary forests of similar ages (i.e., 30–50 years old; Lennox et al. 2018; Matos et al. 2020; Rozendaal et al. 2019). In particular, the dusk chorus patterns of natural regeneration soundscapes, which were heavily degraded in pastures, were nearly identical to reference forests (Figure 4). The discrepancy between the strong recovery of the dusk chorus and a relative lack of recovery during the dawn chorus may indicate that natural regeneration sites still lack aspects of species diversity/abundance that generate these patterns in reference forests. Alternatively, it could indicate that fauna within naturally regenerated forests, which may be in closer proximity to human activity than protected areas, have temporally shifted their behavioral patterns to avoid human activity and infrastructure noise (Pease and Gilbert 2025). These patterns reflect the need for acoustic comparisons to be underpinned by strong methodological choices and for acoustic indices to be applied in proper contexts (Bradfer‐Lawrence et al. 2023). Comparing soundscapes only during the dawn chorus, as many previous studies have done (Buxton et al. 2018; Darras et al. 2025), may underestimate the evidence of ecological recovery.

In contrast to the patterns observed in natural regenerating forests, plantations had significantly lower similarities to reference forests (Figure 4a). Plantations also displayed consistently depressed levels of acoustic energy, suggesting lower levels of acoustic recovery, possibly due to the lack of key components of native biodiversity (Felton et al. 2010; Healey and Gara 2003; Wang et al. 2022; Figure 3a). Such defaunation effects have often accompanied the use of exotic tree species in forest plantations (Tudge et al. 2023; Wang et al. 2022). The observed results highlight the potential advantages to the PES of natural regeneration from an ecological restoration perspective (Crouzeilles et al. 2017) in certain contexts. Interestingly, plantations and natural regeneration sites did not significantly differ in their similarity to pastures, suggesting that plantations have a unique ecological trajectory that does not resemble natural forest ecosystems but is ecologically distinct from pastures (Felton et al. 2010). However, it is important to note that our measure of soundscape similarity is insensitive to direction (e.g., louder or quieter). Therefore, while similarity values between the two PES types and pastures may not have differed significantly, the differences may emerge from distinct underlying ecological patterns. Indeed, natural regeneration sites averaged louder than pastures, while plantations consistently exhibited lower acoustic energy (Figure 3a).

Research suggests that biodiversity within plantations could be improved with different management decisions (Hartley 2002) such as the planting of mixed and native species (Wang et al. 2022). All the plantations in our study (and indeed most within the PES) were monocultures of non‐native species planted in relatively short (10–20 year) harvesting cycles. This lack of structural complexity and relatively younger age may contribute to the observed patterns and earlier stages of recovery.

### 4.4 Implications for the PES Program

While we could not explicitly test whether the soundscape changes described here would have occurred without the introduction of the PES, multiple studies have found the program to have contributed to forest regrowth, land abandonment and to the restoration of forests on previously cleared pastures across Costa Rica and specifically in the Nicoya peninsula (Arroyo‐Mora et al. 2005; Calvo‐Alvarado et al. 2009; Daniels et al. 2010; Sierra and Russman 2006). We conducted robustness checks by matching PES sites to controls using meta‐data known to impact forest cover and recovery (specifically slope and distance to roads; Figure S5) and revealed patterns largely consistent with our overall analysis. Taken together, these data suggest that the PES program has successfully contributed to the improvement of some aspects of biodiversity and driven ecological restoration, especially in naturally regenerating forests. We believe this represents some of the most robust evidence to date that wealth redistribution and ecosystem restoration initiatives can benefit biodiversity on large spatial scales.

While various policy mechanisms have been implemented to finance the conservation and restoration of nature, few have proved both sustainable and effective in reducing environmental damage. Costa Rica is one of the few countries to halt and reverse deforestation by, alongside other regulations and socio‐economic conditions (Sánchez‐Azofeifa et al. 2007), establishing the world's first national‐scale PES program in 1996 (Pagiola 2008). The PES now protects over 200,000 ha with the specific goals of recognizing the water provisioning, carbon storage, biodiversity protection, and scenic “beauty” of the nation's forests (Sánchez‐Azofeifa et al. 2007). While the financial scale of program subsidies (Chapman et al. 2020; Fletcher and Breitling 2012) is limited (~70 USD/ha/year), and research has found mixed effects on poverty alleviation (Arriagada et al. 2012; Lansing 2014), the program represents a unique national‐scale experiment to evaluate the environmental impacts of distributing wealth directly to local land stewards.

Today, the PES program is widely regarded as a model program and an integral part of Costa Rica's progress in protecting its natural heritage. Having recognized the link between ecological degradation and inequality, the program is built on the need to address the social challenges that underpin environmental destruction at scale. Weaknesses in the PES program remain, and improvements are needed to achieve truly equitable wealth redistribution, but studies suggest that many program participants perceive strong socio‐economic benefits (Cole 2010). Costa Rica's attempt to distribute wealth directly to those living in association with nature—through taxes on environmentally destructive behavior and rewards for ecological stewardship—represents a model that could be replicated and expanded globally. Learning from attempts to improve Costa Rica's PES (Barton et al. 2009) is especially relevant given both increasing environmental degradation and economic inequality in the 21st century.

## 5 Conclusion

We demonstrate large‐scale ecological recovery across Payment for Ecosystem Services (PES) sites in Costa Rica's Nicoya Peninsula, following 27 years of the national PES program. Costa Rica's PES sites collectively cover over 200,000 ha, and our regional findings may be indicative of ecological recovery at a broader, national scale. Naturally regenerated forests on private lands enrolled in the PES can harbor acoustic signatures that are similar to those of protected areas, while areas reforested with monocultures share less acoustic similarity to protected forests. Future research will be needed to examine the temporal dynamics and context‐dependency of these effects, and test whether similar programs have the potential to help reverse the loss of biodiversity in regions across the globe. Nevertheless, our research emphasizes that, by prioritizing the redistribution of wealth alongside nature protection, local communities can enable biodiversity recovery at massive spatial scales. Our research adds to a growing body of literature that highlights the tangible links between social equity and the health of ecosystems across landscapes. Embracing these solutions to restore biosphere integrity unlocks the possibility to simultaneously address the challenges of biodiversity loss, climate change and global inequality.

## Author Contributions

G.L.D. devised the study, carried out the fieldwork, collaborated on the data analysis and wrote the manuscript. J.H. heavily contributed to the data analysis, index creation and provided feedback on the manuscript. D.H.D. and T.B.‐L. helped shape the analytical approach and improved various elements of the manuscript. L.K.W., R.C., and Y.L. provided substantial feedback on the manuscript during multiple rounds of internal revisions. C.D.Q., J.‐A.J.F., A.M.R., E.M.S., G.N.C., M.C., and I.S.P. provided essential support in ensuring the research was relevant for local stakeholders, securing permits, extracting relevant information from government databases, and executing the fieldwork in Costa Rica. L.V. carried out the manual annotations. T.W.C. guided the development of the project and collaborated closely with G.L.D. in the writing of the manuscript.

## Conflicts of Interest

The authors declare no conflicts of interest.

## Supporting information


**Appendix S1:** gcb70730‐sup‐0001‐Supinfo.zip.

## Data Availability

All data is available to view and download from the ETH data library platform at the following link: https://www.research‐collection.ethz.ch/handle/20.500.11850/680152. All the code used for the analyses are available to view and use in the following public repository https://github.com/GiacomoLD97/Costa_Rica_PSA_Acoustic_Analysis.

## References

[gcb70730-bib-0084] Akaike, H. 1987. “Factor Analysis and AIC.” Psychometrika 52: 317–332.

[gcb70730-bib-0001] Alcocer, I. , H. Lima , L. S. M. Sugai , and D. Llusia . 2022. “Acoustic Indices as Proxies for Biodiversity: A Meta‐Analysis.” Biological Reviews 97, no. 6: 2209–2236. 10.1111/brv.12890. 35978471 PMC9804652

[gcb70730-bib-0002] Andam, K. S. , P. J. Ferraro , K. R. E. Sims , A. Healy , and M. B. Holland . 2010. “Protected Areas Reduced Poverty in Costa Rica and Thailand.” Proceedings of the National Academy of Sciences of the United States of America 107, no. 22: 9996–10001. 10.1073/pnas.0914177107. 20498058 PMC2890456

[gcb70730-bib-0003] Arriagada, R. A. , P. J. Ferraro , E. O. Sills , S. K. Pattanayak , and S. Cordero‐Sancho . 2012. “Do Payments for Environmental Services Affect Forest Cover? A Farm‐Level Evaluation From Costa Rica.” Land Economics 88, no. 2: 382–399. 10.3368/le.88.2.382.

[gcb70730-bib-0004] Arroyo‐Mora, J. P. , G. A. Sánchez‐Azofeifa , B. Rivard , J. C. Calvo , and D. H. Janzen . 2005. “Dynamics in Landscape Structure and Composition for the Chorotega Region, Costa Rica From 1960 to 2000.” Agriculture, Ecosystems & Environment 106, no. 1: 27–39. 10.1016/j.agee.2004.07.002.

[gcb70730-bib-0005] Barfield, R. J. , P. Auerbach , L. A. Geyer , and T. K. McIntosh . 1979. “Ultrasonic Vocalizations in Rat Sexual Behavior.” American Zoologist 19, no. 2: 469–480.

[gcb70730-bib-0006] Barton, D. N. , D. P. Faith , G. M. Rusch , H. Acevedo , L. Paniagua , and M. Castro . 2009. “Environmental Service Payments: Evaluating Biodiversity Conservation Trade‐Offs and Cost‐Efficiency in the Osa Conservation Area, Costa Rica.” Journal of Environmental Management 90, no. 2: 901–911. 10.1016/j.jenvman.2008.02.010. 18423841

[gcb70730-bib-0007] Beecher, M. D. , and E. A. Brenowitz . 2005. “Functional Aspects of Song Learning in Songbirds.” Trends in Ecology & Evolution 20, no. 3: 143–149. 10.1016/j.tree.2005.01.004. 16701358

[gcb70730-bib-0008] Boelman, N. T. , G. P. Asner , P. J. Hart , and R. E. Martin . 2007. “Multi‐Trophic Invasion Resistance in Hawaii: Bioacoustics, Field Surveys, and Airborne Remote Sensing.” Ecological Applications 17, no. 8: 2137–2144. 10.1890/07-0004.1. 18213957

[gcb70730-bib-0009] Boyce, J. K. 1994. “Inequality as a Cause of Environmental Degradation.” Ecological Economics 11, no. 3: 169–178. 10.1016/0921-8009(94)90198-8.

[gcb70730-bib-0010] Bradfer‐Lawrence, T. , Z. Buřivalová , and D. H. Dent . 2025. “Deriving Meaning From Acoustic Data in Hyper‐Diverse Ecosystems.” Trends in Ecology & Evolution 40: 431–435. 10.1016/j.tree.2025.03.004. 40221278

[gcb70730-bib-0011] Bradfer‐Lawrence, T. , C. Desjonqueres , A. Eldridge , A. Johnston , and O. Metcalf . 2023. “Using Acoustic Indices in Ecology: Guidance on Study Design, Analyses and Interpretation.” Methods in Ecology and Evolution 14, no. 9: 2192–2204. 10.1111/2041-210X.14194.

[gcb70730-bib-0012] Bradfer‐Lawrence, T. , B. Duthie , C. Abrahams , et al. 2025. “The Acoustic Index User's Guide: A Practical Manual for Defining, Generating and Understanding Current and Future Acoustic Indices.” Methods in Ecology and Evolution 16, no. 6: 1040–1050. 10.1111/2041-210X.14357.

[gcb70730-bib-0013] Bradfer‐Lawrence, T. , N. Gardner , L. Bunnefeld , N. Bunnefeld , S. G. Willis , and D. H. Dent . 2019. “Guidelines for the Use of Acoustic Indices in Environmental Research.” Methods in Ecology and Evolution 10, no. 10: 1796–1807. 10.1111/2041-210X.13254.

[gcb70730-bib-0014] Brancalion, P. H. S. , F. Hua , F. H. Joyce , A. Antonelli , and K. D. Holl . 2025. “Moving Biodiversity From an Afterthought to a Key Outcome of Forest Restoration.” Nature Reviews Biodiversity 1: 248–261. 10.1038/s44358-025-00032-1.

[gcb70730-bib-0015] Brown, K. S. 1997. “Diversity, Disturbance, and Sustainable Use of Neotropical Forests: Insects as Indicators for Conservation Monitoring.” Journal of Insect Conservation 1, no. 1: 25–42. 10.1023/A:1018422807610.

[gcb70730-bib-0016] Burivalova, Z. , T. M. Maeda , Y. Purnomo Rayadin , et al. 2022. “Loss of Temporal Structure of Tropical Soundscapes With Intensifying Land Use in Borneo.” Science of the Total Environment 852: 158268. 10.1016/j.scitotenv.2022.158268. 36058325

[gcb70730-bib-0017] Burivalova, Z. , S. Purnomo Orndorff , A. Truskinger , P. Roe , and E. T. Game . 2021. “The Sound of Logging: Tropical Forest Soundscape Before, During, and After Selective Timber Extraction.” Biological Conservation 254: 108812. 10.1016/j.biocon.2020.108812.

[gcb70730-bib-0018] Burivalova, Z. , B. Purnomo Wahyudi , T. M. Boucher , et al. 2019. “Using Soundscapes to Investigate Homogenization of Tropical Forest Diversity in Selectively Logged Forests.” Journal of Applied Ecology 56, no. 11: 2493–2504. 10.1111/1365-2664.13481.

[gcb70730-bib-0019] Buxton, R. T. , M. F. McKenna , M. Clapp , et al. 2018. “Efficacy of Extracting Indices From Large‐Scale Acoustic Recordings to Monitor Biodiversity.” Conservation Biology 32, no. 5: 1174–1184. 10.1111/cobi.13119. 29676813

[gcb70730-bib-0020] Calvo‐Alvarado, J. , B. McLennan , A. Sánchez‐Azofeifa , and T. Garvin . 2009. “Deforestation and Forest Restoration in Guanacaste, Costa Rica: Putting Conservation Policies in Context.” Forest Ecology and Management 258, no. 6: 931–940. 10.1016/j.foreco.2008.10.035.

[gcb70730-bib-0021] Castano Garcia, A. , A. Ambrose , A. Hawkins , and S. Parkes . 2021. “High Consumption, an Unsustainable Habit That Needs More Attention.” Energy Research & Social Science 80: 102241. 10.1016/j.erss.2021.102241.

[gcb70730-bib-0022] Chancel, L. , P. Bothe , and T. Voituriez . 2024. “The Potential of Wealth Taxation to Address the Triple Climate Inequality Crisis.” Nature Climate Change 14, no. 1: 1. 10.1038/s41558-023-01891-2.

[gcb70730-bib-0023] Chapman, M. , T. Satterfield , H. Wittman , and K. M. A. Chan . 2020. “A Payment by Any Other Name: Is Costa Rica's PES a Payment for Services or a Support for Stewards?” World Development 129: 104900. 10.1016/j.worlddev.2020.104900.

[gcb70730-bib-0024] Chen, H. L. , R. L. Lewison , L. An , et al. 2020. “Assessing the Effects of Payments for Ecosystem Services Programs on Forest Structure and Species Biodiversity.” Biodiversity and Conservation 29, no. 7: 2123–2140. 10.1007/s10531-020-01953-3.

[gcb70730-bib-0025] Cheng, Y. , B. Ma , and Y. Sun . 2023. “Does Central Ecological Transfer Payment Enhance Local Environmental Performance? Quasi‐Experimental Evidence From China.” Ecological Economics 212: 107920. 10.1016/j.ecolecon.2023.107920.

[gcb70730-bib-0026] Cole, R. J. 2010. “Social and Environmental Impacts of Payments for Environmental Services for Agroforestry on Small‐Scale Farms in Southern Costa Rica.” International Journal of Sustainable Development and World Ecology 17, no. 3: 208–216. 10.1080/13504501003729085.

[gcb70730-bib-0027] Crouzeilles, R. , M. S. Ferreira , R. L. Chazdon , et al. 2017. “Ecological Restoration Success Is Higher for Natural Regeneration Than for Active Restoration in Tropical Forests.” Science Advances 3, no. 11: e1701345. 10.1126/sciadv.1701345. 29134195 PMC5677348

[gcb70730-bib-0028] Daniels, A. E. , K. Bagstad , V. Esposito , A. Moulaert , and C. M. Rodriguez . 2010. “Understanding the Impacts of Costa Rica's PES: Are We Asking the Right Questions?” Ecological Economics 69, no. 11: 2116–2126. 10.1016/j.ecolecon.2010.06.011.

[gcb70730-bib-0029] Darras, K. F. A. , R. A. Rountree , S. L. Van Wilgenburg , et al. 2025. “Worldwide Soundscapes: A Synthesis of Passive Acoustic Monitoring Across Realms.” Global Ecology and Biogeography 34, no. 5: e70021. 10.1111/geb.70021.

[gcb70730-bib-0030] Farley, J. , and R. Costanza . 2010. “Payments for Ecosystem Services: From Local to Global.” Ecological Economics 69, no. 11: 2060–2068. 10.1016/j.ecolecon.2010.06.010.

[gcb70730-bib-0031] Felton, A. , E. Knight , J. Wood , C. Zammit , and D. Lindenmayer . 2010. “A Meta‐Analysis of Fauna and Flora Species Richness and Abundance in Plantations and Pasture Lands.” Biological Conservation 143, no. 3: 545–554. 10.1016/j.biocon.2009.11.030.

[gcb70730-bib-0032] Fletcher, R. , and J. Breitling . 2012. “Market Mechanism or Subsidy in Disguise? Governing Payment for Environmental Services in Costa Rica.” Geoforum 43, no. 3: 402–411. 10.1016/j.geoforum.2011.11.008.

[gcb70730-bib-0033] Friedman, J. H. 2002. “Stochastic Gradient Boosting.” Computational Statistics & Data Analysis 38, no. 4: 367–378. 10.1016/S0167-9473(01)00065-2.

[gcb70730-bib-0034] Furumo, P. R. , and T. Mitchell Aide . 2019. “Using Soundscapes to Assess Biodiversity in Neotropical Oil Palm Landscapes.” Landscape Ecology 34, no. 4: 911–923. 10.1007/s10980-019-00815-w.

[gcb70730-bib-0035] Harris, J. A. , R. J. Hobbs , E. Higgs , and J. Aronson . 2006. “Ecological Restoration and Global Climate Change.” Restoration Ecology 14, no. 2: 170–176. 10.1111/j.1526-100X.2006.00136.x.

[gcb70730-bib-0036] Hartley, M. J. 2002. “Rationale and Methods for Conserving Biodiversity in Plantation Forests.” Forest Ecology and Management 155, no. 1: 81–95. 10.1016/S0378-1127(01)00549-7.

[gcb70730-bib-0037] Healey, S. P. , and R. I. Gara . 2003. “The Effect of a Teak (*Tectona grandis*) Plantation on the Establishment of Native Species in an Abandoned Pasture in Costa Rica.” Forest Ecology and Management 176, no. 1: 497–507. 10.1016/S0378-1127(02)00235-9.

[gcb70730-bib-0038] Holdridge, L. R. 1967. “Life Zone Ecology.” https://www.cabdirect.org/cabdirect/abstract/19670604180.

[gcb70730-bib-0039] Holland, T. G. , G. D. Peterson , and A. Gonzalez . 2009. “A Cross‐National Analysis of How Economic Inequality Predicts Biodiversity Loss.” Conservation Biology 23, no. 5: 1304–1313. 10.1111/j.1523-1739.2009.01207.x. 19765041

[gcb70730-bib-0040] Ingram, J. C. , D. Wilkie , T. Clements , et al. 2014. “Evidence of Payments for Ecosystem Services as a Mechanism for Supporting Biodiversity Conservation and Rural Livelihoods.” Ecosystem Services 7: 10–21. 10.1016/j.ecoser.2013.12.003.

[gcb70730-bib-0041] IPBES . 2018. “The IPBES Assessment Report on Land Degradation and Restoration.” *Zenodo*. 10.5281/zenodo.3237393.

[gcb70730-bib-0042] Jarrett, D. , R. Barnett , T. Bradfer‐Lawrence , et al. 2025. “Mitigating Bias in Long‐Term Terrestrial Ecoacoustic Studies.” Journal of Applied Ecology 62, no. 4: 761–772. 10.1111/1365-2664.70000.

[gcb70730-bib-0043] Jayachandran, S. , J. de Laat , E. F. Lambin , C. Y. Stanton , R. Audy , and N. E. Thomas . 2017. “Cash for Carbon: A Randomized Trial of Payments for Ecosystem Services to Reduce Deforestation.” Science 357, no. 6348: 267–273. 10.1126/science.aan0568. 28729505

[gcb70730-bib-0044] Kelly, K. G. , C. M. Wood , K. McGinn , et al. 2023. “Estimating Population Size for California Spotted Owls and Barred Owls Across the Sierra Nevada Ecosystem With Bioacoustics.” Ecological Indicators 154: 110851. 10.1016/j.ecolind.2023.110851.

[gcb70730-bib-0045] Knight, E. , T. Rhinehart , D. R. de Zwaan , et al. 2024. “Individual Identification in Acoustic Recordings.” Trends in Ecology & Evolution 39, no. 10: 947–960. 10.1016/j.tree.2024.05.007. 38862357

[gcb70730-bib-0046] Krause, B. 1993. “The Niche Hypothesis.” Soundscape Newsletter 6: 6–10.

[gcb70730-bib-0047] Lansing, D. M. 2014. “Unequal Access to Payments for Ecosystem Services: The Case of Costa Rica.” Development and Change 45, no. 6: 1310–1331. 10.1111/dech.12134.

[gcb70730-bib-0048] Lennox, G. D. , T. A. Gardner , J. R. Thomson , et al. 2018. “Second Rate or a Second Chance? Assessing Biomass and Biodiversity Recovery in Regenerating Amazonian Forests.” Global Change Biology 24, no. 12: 5680–5694. 10.1111/gcb.14443. 30216600

[gcb70730-bib-0049] Löfqvist, S. , F. Kleinschroth , A. Bey , et al. 2023. “How Social Considerations Improve the Equity and Effectiveness of Ecosystem Restoration.” Bioscience 73, no. 2: 134–148. 10.1093/biosci/biac099. 36896142 PMC9991587

[gcb70730-bib-0050] Lundberg, S. , and S.‐I. Lee . 2017. “A Unified Approach to Interpreting Model Predictions (No. arXiv:1705.07874).” *arXiv*. http://arxiv.org/abs/1705.07874.

[gcb70730-bib-0051] MacGregor‐Fors, I. , and J. E. Schondube . 2011. “Use of Tropical Dry Forests and Agricultural Areas by Neotropical Bird Communities.” Biotropica 43, no. 3: 365–370. 10.1111/j.1744-7429.2010.00709.x.

[gcb70730-bib-0052] Marín‐Gómez, O. H. , W. Dáttilo , J. R. Sosa‐López , D. Santiago‐Alarcon , and I. MacGregor‐Fors . 2020. “Where Has the City Choir Gone? Loss of the Temporal Structure of Bird Dawn Choruses in Urban Areas.” Landscape and Urban Planning 194: 103665. 10.1016/j.landurbplan.2019.103665.

[gcb70730-bib-0053] Marten, K. , and P. Marler . 1977. “Sound Transmission and Its Significance for Animal Vocalization.” Behavioral Ecology and Sociobiology 2, no. 3: 271–290. 10.1007/BF00299740.

[gcb70730-bib-0054] Martin, A. , N. Gross‐Camp , B. Kebede , and S. McGuire . 2014. “Measuring Effectiveness, Efficiency and Equity in an Experimental Payments for Ecosystem Services Trial.” Global Environmental Change 28: 216–226. 10.1016/j.gloenvcha.2014.07.003. 28149003 PMC5268343

[gcb70730-bib-0055] Matos, F. A. R. , L. F. S. Magnago , C. Aquila Chan Miranda , et al. 2020. “Secondary Forest Fragments Offer Important Carbon and Biodiversity Cobenefits.” Global Change Biology 26, no. 2: 509–522. 10.1111/gcb.14824. 31486174

[gcb70730-bib-0056] Maya‐Elizarrarás, E. , and J. E. Schondube . 2015. “Birds, Cattle, and Bracken Ferns: Bird Community Responses to a Neotropical Landscape Shaped by Cattle Grazing Activities.” Biotropica 47, no. 2: 236–245. 10.1111/btp.12196.

[gcb70730-bib-0057] Metcalf, O. C. , J. Barlow , C. Devenish , S. Marsden , E. Berenguer , and A. C. Lees . 2021. “Acoustic Indices Perform Better When Applied at Ecologically Meaningful Time and Frequency Scales.” Methods in Ecology and Evolution 12, no. 3: 421–431. 10.1111/2041-210X.13521.

[gcb70730-bib-0058] Miyamoto, M. 2020. “Poverty Reduction Saves Forests Sustainably: Lessons for Deforestation Policies.” World Development 127: 104746. 10.1016/j.worlddev.2019.104746.

[gcb70730-bib-0059] Müller, J. , O. Mitesser , H. M. Schaefer , et al. 2023. “Soundscapes and Deep Learning Enable Tracking Biodiversity Recovery in Tropical Forests.” Nature Communications 14, no. 1: 1. 10.1038/s41467-023-41693-w. PMC10582010 37848442

[gcb70730-bib-0085] Pagiola, S. 2008. “Payments for Environmental Services in Costa Rica.” Ecological Economics 65, no. 4: 712–724. 10.1016/j.ecolecon.2007.07.033.

[gcb70730-bib-0060] Paiva, I. G. , A. M. Auad , B. A. Veríssimo , and L. C. P. Silveira . 2020. “Differences in the Insect Fauna Associated to a Monocultural Pasture and a Silvopasture in Southeastern Brazil.” Scientific Reports 10, no. 1: 12112. 10.1038/s41598-020-68973-5. 32694546 PMC7374564

[gcb70730-bib-0061] Pease, B. S. , and N. A. Gilbert . 2025. “Light Pollution Prolongs Avian Activity.” Science 389, no. 6762: 818–821. 10.1126/science.adv9472. 40839731

[gcb70730-bib-0062] Pijanowski, B. C. , L. J. Villanueva‐Rivera , S. L. Dumyahn , et al. 2011. “Soundscape Ecology: The Science of Sound in the Landscape.” Bioscience 61, no. 3: 203–216. 10.1525/bio.2011.61.3.6.

[gcb70730-bib-0063] Pulsford, S. A. , P. S. Barton , D. A. Driscoll , and D. B. Lindenmayer . 2019. “Interactive Effects of Land Use, Grazing and Environment on Frogs in an Agricultural Landscape.” Agriculture, Ecosystems & Environment 281: 25–34. 10.1016/j.agee.2019.05.003.

[gcb70730-bib-0064] Ribeiro, J. W. , K. Harmon , G. A. Leite , T. N. de Melo , J. LeBien , and M. Campos‐Cerqueira . 2022. “Passive Acoustic Monitoring as a Tool to Investigate the Spatial Distribution of Invasive Alien Species.” Remote Sensing 14, no. 18: 18. 10.3390/rs14184565.

[gcb70730-bib-0065] Romanelli, J. P. , P. Meli , J. P. B. Santos , et al. 2022. “Biodiversity Responses to Restoration Across the Brazilian Atlantic Forest.” Science of the Total Environment 821: 153403. 10.1016/j.scitotenv.2022.153403. 35101503

[gcb70730-bib-0066] Rozendaal, D. M. A. , F. Bongers , T. M. Aide , et al. 2019. “Biodiversity Recovery of Neotropical Secondary Forests.” Science Advances 5, no. 3: eaau3114. 10.1126/sciadv.aau3114. 30854424 PMC6402850

[gcb70730-bib-0067] Sader, S. A. , and A. T. Joyce . 1988. “Deforestation Rates and Trends in Costa Rica, 1940 to 1983.” Biotropica 20, no. 1: 11–19. 10.2307/2388421.

[gcb70730-bib-0068] Samii, C. , M. Lisiecki , P. Kulkarni , et al. 2014. “Effects of Payment for Environmental Services (PES) on Deforestation and Poverty in Low and Middle Income Countries: A Systematic Review.” Campbell Systematic Reviews 10, no. 1: 1–95. 10.4073/csr.2014.11.

[gcb70730-bib-0069] Sánchez‐Azofeifa, G. A. , A. Pfaff , J. A. Robalino , and J. P. Boomhower . 2007. “Costa Rica's Payment for Environmental Services Program: Intention, Implementation, and Impact.” Conservation Biology 21, no. 5: 1165–1173. 10.1111/j.1523-1739.2007.00751.x. 17883482

[gcb70730-bib-0070] Searcy, W. A. , R. C. Anderson , and S. Nowicki . 2006. “Bird Song as a Signal of Aggressive Intent.” Behavioral Ecology and Sociobiology 60, no. 2: 234–241. 10.1007/s00265-006-0161-9.

[gcb70730-bib-0071] Sethi, S. S. , A. Bick , R. M. Ewers , et al. 2023. “Limits to the Accurate and Generalizable Use of Soundscapes to Monitor Biodiversity.” Nature Ecology & Evolution 7, no. 9: 1373–1378. 10.1038/s41559-023-02148-z. 37524796 PMC10482675

[gcb70730-bib-0072] Sierra, R. , and E. Russman . 2006. “On the Efficiency of Environmental Service Payments: A Forest Conservation Assessment in the Osa Peninsula, Costa Rica.” Ecological Economics 59, no. 1: 131–141. 10.1016/j.ecolecon.2005.10.010.

[gcb70730-bib-0073] Sueur, J. , and A. Farina . 2015. “Ecoacoustics: The Ecological Investigation and Interpretation of Environmental Sound.” Biosemiotics 8, no. 3: 493–502. 10.1007/s12304-015-9248-x.

[gcb70730-bib-0074] Sueur, J. , S. Pavoine , O. Hamerlynck , and S. Duvail . 2008. “Rapid Acoustic Survey for Biodiversity Appraisal.” PLoS One 3, no. 12: e4065. 10.1371/journal.pone.0004065. 19115006 PMC2605254

[gcb70730-bib-0075] Towsey, M. 2017. “The Calculation of Acoustic Indices Derived From Long‐Duration Recordings of the Natural Environment.” https://www.semanticscholar.org/paper/The‐calculation‐of‐acoustic‐indices‐derived‐from‐of‐Towsey/cd3fe08d225da2b2826d75bd62bb715eb2a0ae01.

[gcb70730-bib-0076] Tudge, S. J. , Z. M. Harris , R. J. Murphy , A. Purvis , and A. De Palma . 2023. “Global Trends in Biodiversity With Tree Plantation Age.” Global Ecology and Conservation 48: e02751. 10.1016/j.gecco.2023.e02751.

[gcb70730-bib-0077] Vaz, E. , A. Anthony , and M. McHenry . 2017. “The Geography of Environmental Injustice.” Habitat International 59: 118–125. 10.1016/j.habitatint.2016.12.001.

[gcb70730-bib-0078] Wang, C. , W. Zhang , X. Li , and J. Wu . 2022. “A Global Meta‐Analysis of the Impacts of Tree Plantations on Biodiversity.” Global Ecology and Biogeography 31, no. 3: 576–587. 10.1111/geb.13440.

[gcb70730-bib-0079] Wiik, E. , R. d'Annunzio , E. Pynegar , D. Crespo , N. Asquith , and J. P. G. Jones . 2019. “Experimental Evaluation of the Impact of a Payment for Environmental Services Program on Deforestation.” Conservation Science and Practice 1, no. 2: e8. 10.1111/csp2.8.

[gcb70730-bib-0080] Wood, C. M. , V. D. Popescu , H. Klinck , et al. 2019. “Detecting Small Changes in Populations at Landscape Scales: A Bioacoustic Site‐Occupancy Framework.” Ecological Indicators 98: 492–507. 10.1016/j.ecolind.2018.11.018.

[gcb70730-bib-0081] Yoh, N. , W. Mbamy , B. L. Gottesman , et al. 2024. “Impacts of Logging, Hunting, and Conservation on Vocalizing Biodiversity in Gabon.” Biological Conservation 296: 110726. 10.1016/j.biocon.2024.110726.

[gcb70730-bib-0082] Zhen, N. , B. Fu , Y. Lu , and S. Wang . 2014. “Poverty Reduction, Environmental Protection and Ecosystem Services: A Prospective Theory for Sustainable Development.” Chinese Geographical Science 24, no. 1: 83–92. 10.1007/s11769-014-0658-5.

[gcb70730-bib-0083] Znidersic, E. , and D. M. Watson . 2022. “Acoustic Restoration: Using Soundscapes to Benchmark and Fast‐Track Recovery of Ecological Communities.” Ecology Letters 25, no. 7: 1597–1603. 10.1111/ele.14015. 35474408 PMC9321842

