# Supplementary material for: Large‐Scale Forest Restoration Accompanied by Biodiversity Recovery in Costa Rica's Redistributive Payment for Ecosystem Service Program
Source: Glob Chang Biol. 2026 Feb 4;32(2):e70730. doi: 10.1111/gcb.70730 (PMC12869352; doi:10.1111/gcb.70730)
Supplement: Supplementary file 1 — Appendix S1: gcb70730‐sup‐0001‐Supinfo.zip. [file GCB-32-e70730-s001.zip › gcb70730-sup-0001-FigureS1-S5-TableS1-S5@Supplementary Information.pdf]

Supplementary information

Additional Methods

Fieldwork

Working with FONAFIFO and filtering active PES sites according to our requirements originally produced a list of 114 sites of three PES contract types: 67 “Protection of Forests”, 41 “Reforestation” and 6 “Regeneration”. We eliminated the “Regeneration” type as there were insufficient sites to provide a proper comparison. To ensure that our sites were representative of the climactic variation across the Nicoya Peninsula we identified nine identified nine distinct geographic and climatic areas, from which we aimed to sample roughly equal numbers of contracts and land use types. We sampled 50 of the 67 natural regeneration (Protection of forest) sites provided by FONAFIFO. Only 41 plantations sites originally matched our selection requirements, so we relaxed the age requirement to a minimum of seven years in order to reach our final count of 51 sites. Of the protected areas we sampled, the first was integrated into the national system in 1974 (Barra Honda National Park), while the last was integrated in 2009 (Wildlife Refuge Conchal).

We used audiomoth hardware for its low-cost and open-source design, which would allow other projects to replicate our methods with relatively low levels of up-front investment.

We did not directly measure the distance to the nearest road when placing recorders. Instead, we extracted estimates from “Openstreetmap”, these values are listed in Supplementary Table 1. Acoustic interference from roads should not have affected the outcomes of the analysis presented here, especially as such sounds rarely exceeded the 1 kHz threshold, which we later exclude (see below). Recordings were only started once all other field data had been recorded, to avoid capturing the sound of fieldwork.

Analysis

Gradient boosted machines are ensemble variants of random forest models capable of handling highly complex data. SHAP analyses are useful for investigating the decision making of machine learning models. By iteratively considering all combinations of variables and their effect on the model, a value is assigned to each variable, which represents the magnitude and direction (positive or negative) of its impact on model predictions. The remaining data enables strong paired comparisons among sites as (acoustic) differences driven by climate are accounted for.

Using an independent training set of 20 minutes a lab technician (LV) was trained by authors with extensive field experience in the tropics (GLD, LW) to identify sounds as having been produced by amphibians, birds, insects, or mammals (split into wild, domestic, and human). The lab technician then annotated the 120 randomly selected minutes.

The Wasserstein distance, or earth-movers distance, considers two distributions and calculates the minimum ‘cost’ of moving one into the other, taken to be the amount of ‘earth’ multiplied by the distance moved. The distributions of land-use types are made up of ΣPMN summed across 1 kHz frequency bands within sites and then averaged across the 10-minute range of interest. This results in a separate distribution of points = $(10 \times \text{number of sites})$

for each time-frequency intersection. The time-frequency specificity of these distance calculations is essential to create relevant ecological comparisons.

Figures

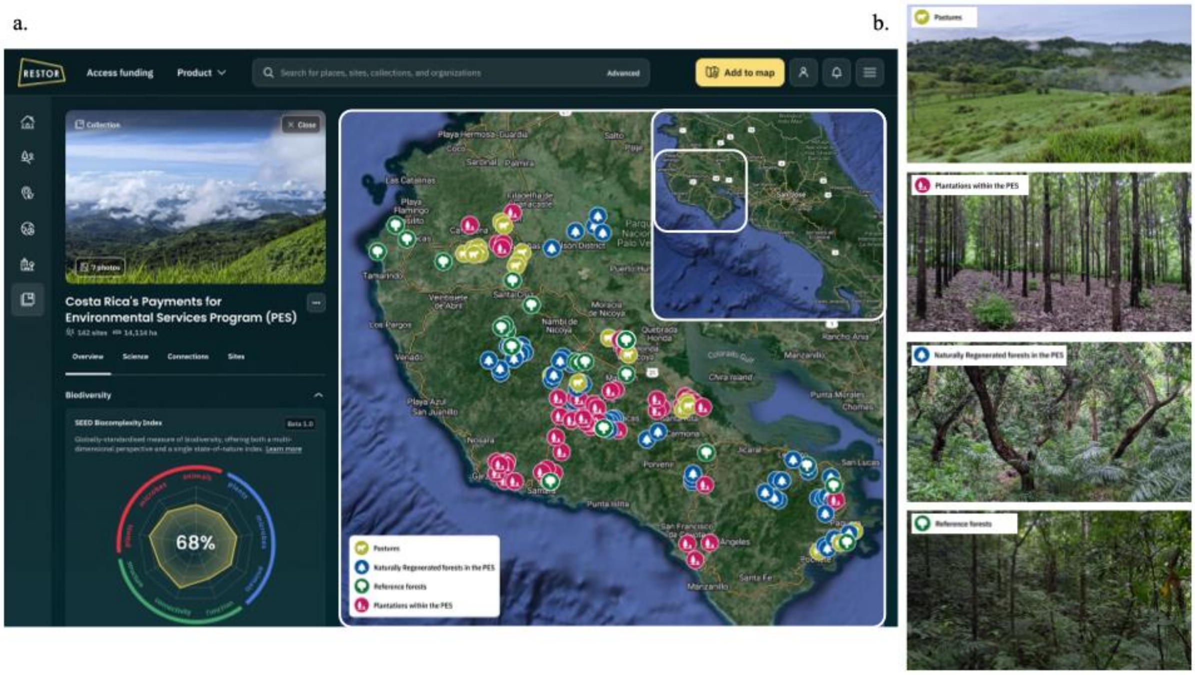

Supplementary Figure 1: The study area in Costa Rica (A) A map of all the recording locations in the Nicoya Peninsula as seen on RESTOR, an online open-access platform for conservation and restoration. Each point represents a single sampling location. This map can be found and interacted with on www.restor.eco (B) Example photos of each land-use type taken from the field during the sampling period in 2022.

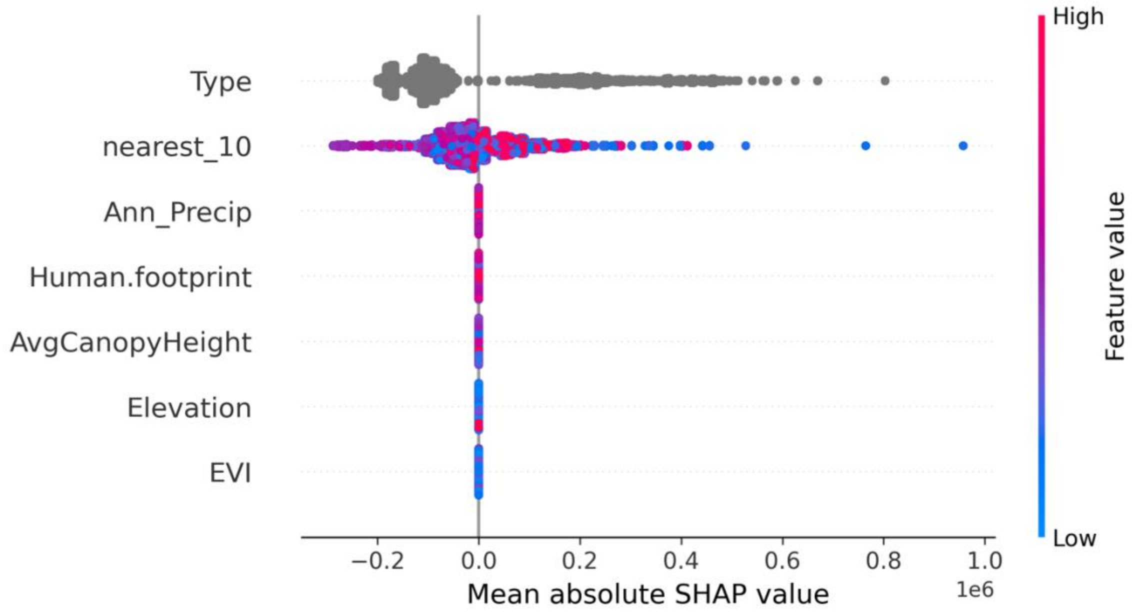

Supplementary Figure 2: Establishing the utility of $\sum PMN$. SHAP values indicate each variable's effect on model (Gradient boosted machine) predictions. Mean SHAP values, plotted along the x axis, indicate how each individual datapoint influences model outputs, with larger absolute values indicating larger magnitudes and signs indicating the direction of effect. Additionally, each datapoint is colored by its value relative to the mean of its respective variable (e.g., Time bins directly after midnight are "low" and colored blue, while time bins directly before midnight are "high" and colored red). Results indicate land-use type is the strongest predictor for variations in ΣPMN, suggesting our index captures habitat-specific acoustic dynamics.

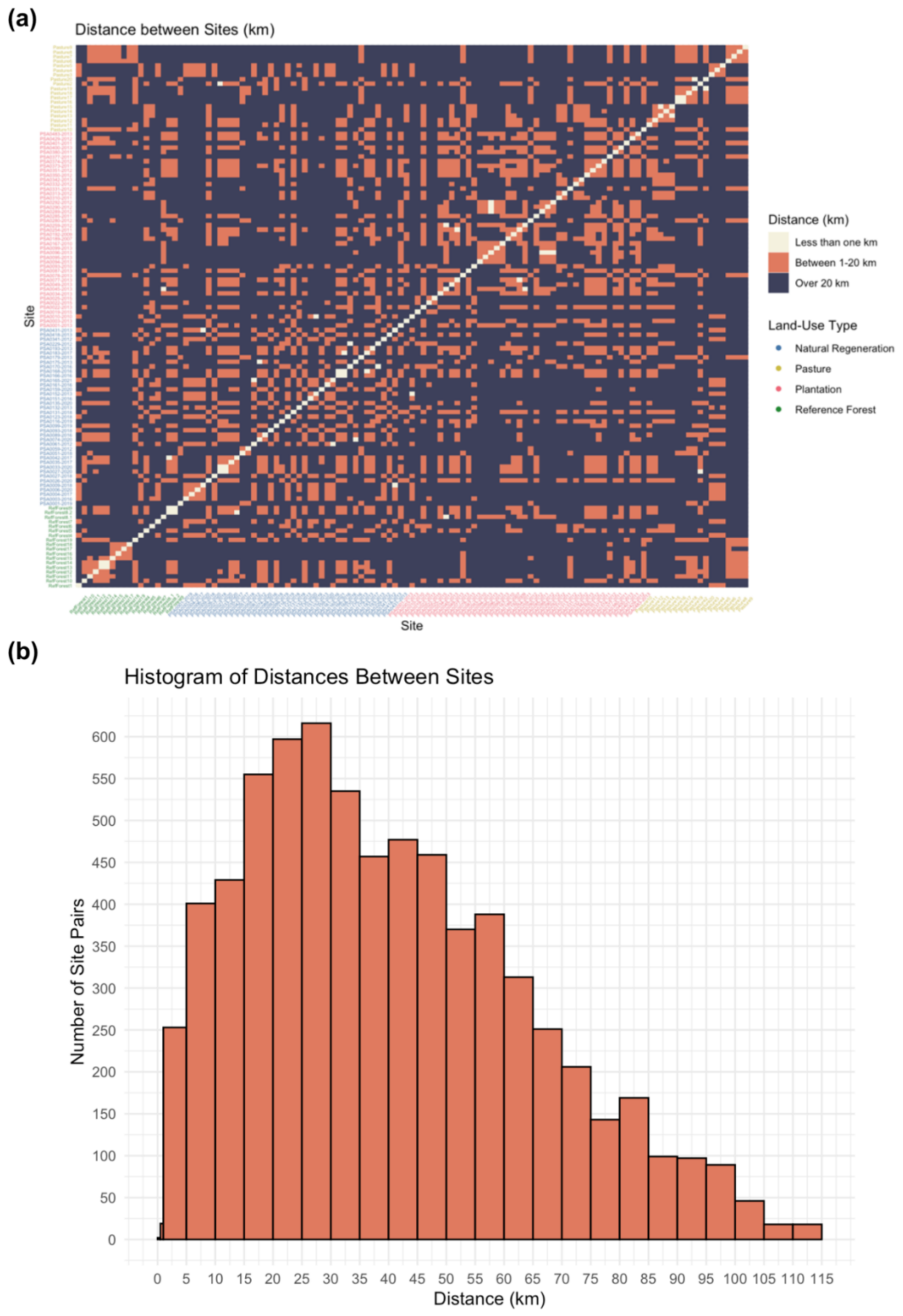

Supplementary Figure 3: Physical distances between sites. (A) A heatmap depicting the physical distances between each pair of sites, broken down into three categories; less than one km, between 1 and 20 km, and over 20km. Site labels are colored by their associated land-use type. (B) A histogram shows the distribution of physical distances between every unique pair of sites. The first two breaks in the histogram are for 0.5 km and 1km, after which breaks are at multiples of 5km.

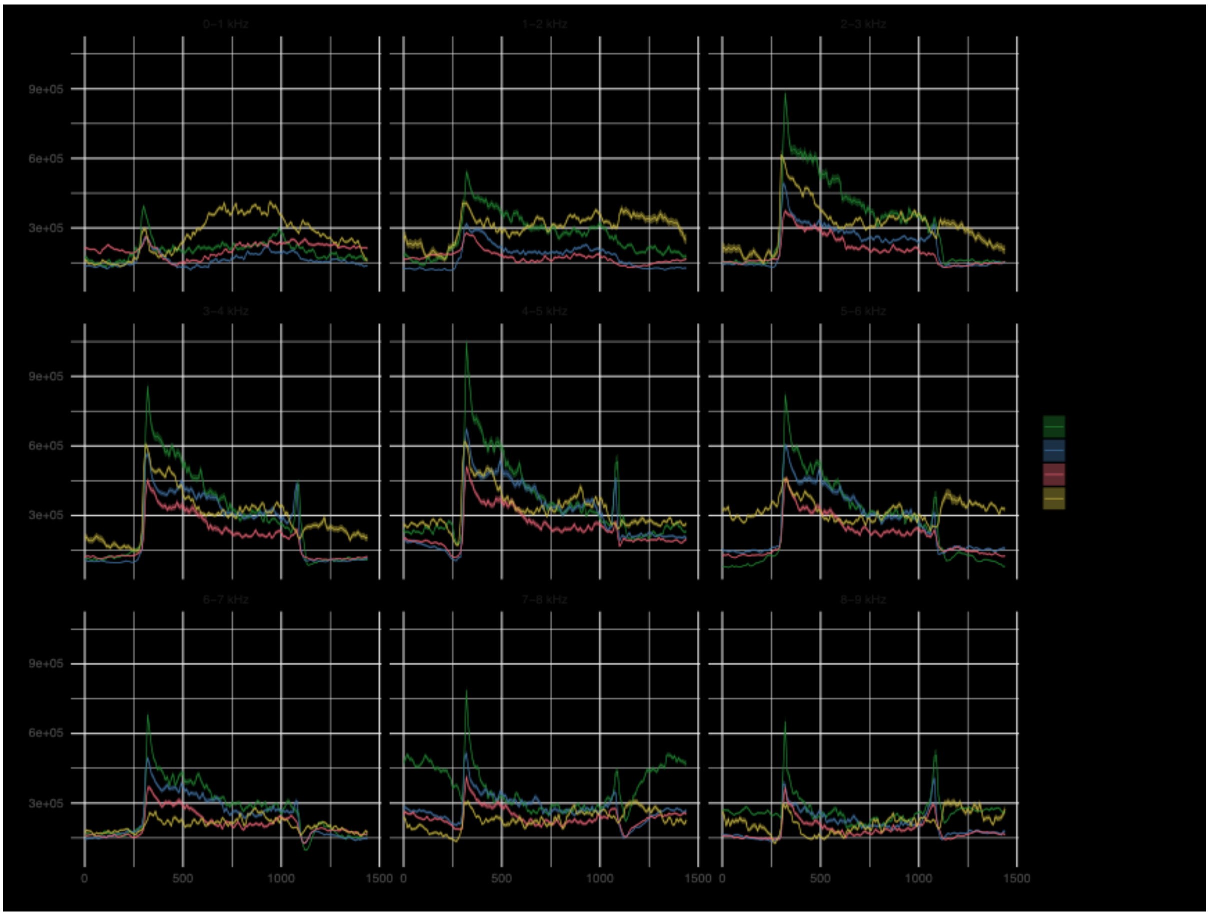

Supplementary Figure 4: Acoustic Patterns by Frequency Band. Average daily ΣPMN patterns for each land-use type are plotted, disaggregated into 1 kHz bands from 0-9 kHz. Shaded colors around lines indicate standard errors of the means ($SEM = \frac{SD}{\sqrt{N}}$).

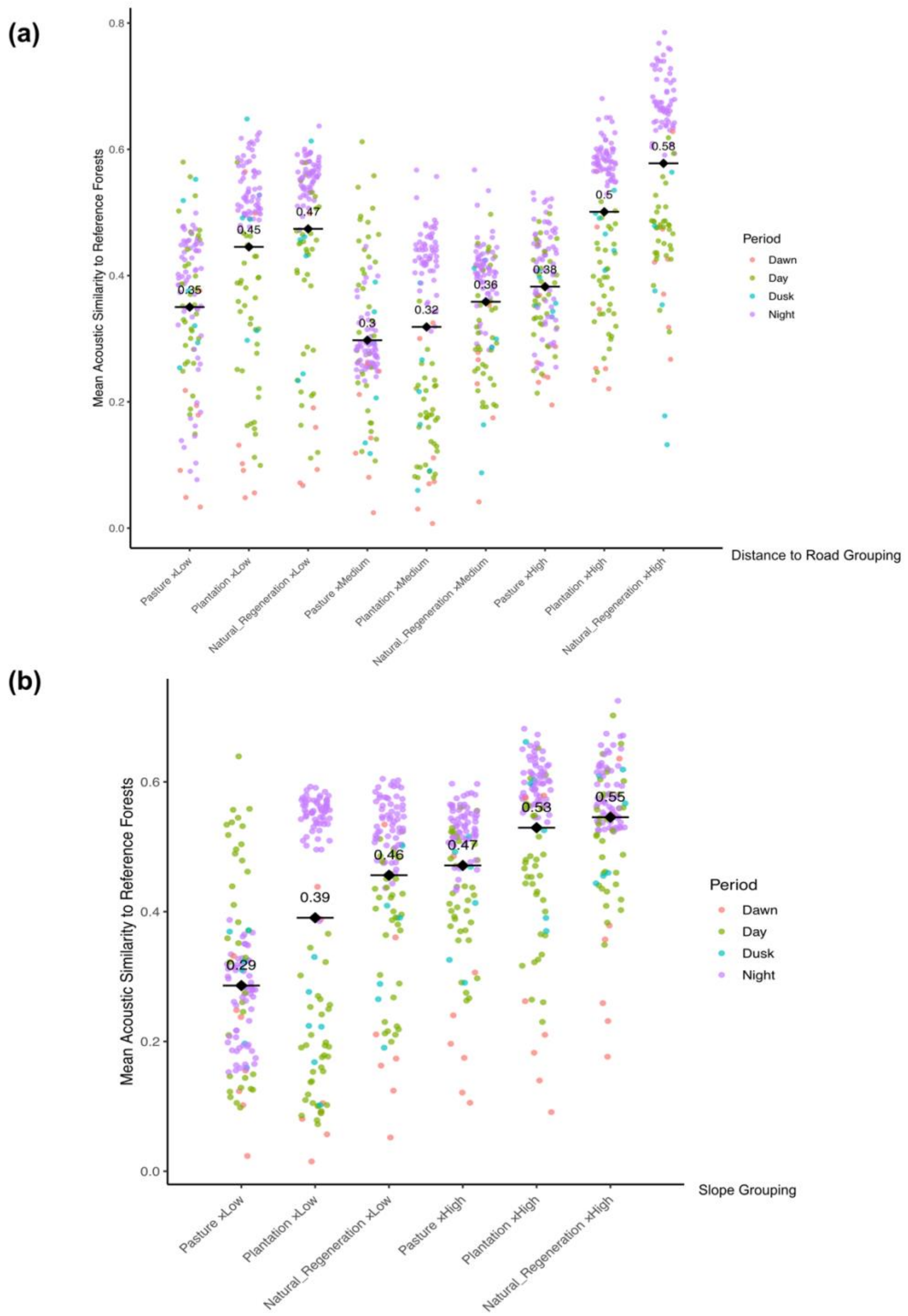

Supplementary Figure 5: Like-for-like comparisons suggest PES contribution to recovery. Definitively establishing the causality of observed changes to a specific policy is challenging. To confirm the validity of our results we re-ran our analysis on sites matches by meta-data characteristics known to be important for land-use management decisions and forest cover. Results of our acoustic distance calculations to reference forests are shown for the other three land use classes, every point represents an average similarity value for a 10-minute and 1 kHz time-frequency intersection, colored by the period of the day. Sites were grouped by (A) Distance to roads or (B) Slope.

Tables

Supplementary Table 1 is provided as a seperate csv file.

Supplementary Table 2: Acoustic Distance calculations using all recorded times overestimates recovery.

Site Type	Distance to Pasture	Similarity to Pasture	Post-Hoc Test	Distance to Reference Forest	Distance to Reference Forest	Post-Hoc Test
Reference Forests	105,405	0.349	****	0	1.0	
Natural Regeneration (PES)	93,573	0.435	*	72,774	0.537	****
Monoculture Plantations (PES)	100,564	0.415	****	97,806	0.455	****
Pastures	0	1.0		105,405	0.349	****
Results of Statistical Tests	Q = 11.3<br>*p* = 0.00352	Q = 11.3<br>*p* = 0.00352		Q = 53<br>*p* < 0.0001	Q = 46.8<br>*p* < 0.001	

Supplementary Table 2:

Results of the acoustic distance (and scaled similarity values) comparisons within all recorded times between every site type and the two baseline ecosystems – Reference Forests and Pastures. Distance and similarity values are averaged across all tested bins. Percentages in the final column are calculated as average of the percentage skew towards either baseline in each matching bin. Results of Post-hoc tests between groups are indicated by brackets where *ns* = not significant and **** indicates a *p* value of < 0.0001.

Supplementary Table 3: Acoustic Distance calculations during the dawn chorus underestimates recovery.

Site Type	Distance to Pasture	Similarity to Pasture	Post-Hoc Test	Distance to Reference Forest	Distance to Reference Forest	Post-Hoc Test
Reference Forests	189,191	0.151	**** (Reference Forests vs Natural Regeneration); **** (Reference Forests vs Monoculture Plantations)	0	1.0	
Natural Regeneration (PES)	110,453	0.373	**** (Natural Regeneration vs Monoculture Plantations)	143,595	0.298	**** (Natural Regeneration vs Monoculture Plantations); **** (Natural Regeneration vs Pastures)
Monoculture Plantations (PES)	124,810	0.335		206,538	0.184	**** (Monoculture Plantations vs Pastures)
Pastures	0	1.0		189,191	0.151	
Results of Statistical Tests	Q = 20<br>$p < 0.001$	Q = 20<br>$p < 0.001$		Q = 14.6<br>$p < 0.0001$	Q = 14.6<br>$p < 0.001$	

Supplementary Table 3:

Results of the acoustic distance (and scaled similarity values) comparisons during the dawn chorus between every site type and the two baseline ecosystems – Reference Forests and Pastures. Distance and similarity values are averaged across all tested bins. Percentages in the final column are calculated as average of the percentage skew towards either baseline in each matching bin. Results of Post-hoc tests between groups are indicated by brackets where *ns* = not significant and **** indicates a p value of < 0.0001.

Supplementary Table 4: Acoustic Distance calculations using all frequency bands overestimates recovery

Site Type	Distance to Pasture	Similarity to Pasture	Post-Hoc Test	Distance to Reference Forest	Distance to Reference Forest	Post-Hoc Test
Reference Forests	106,190	0.346	****	0	1.0	
Natural Regeneration (PES)	96,172	0.434	****	70,205	0.554	****
Monoculture Plantations (PES)	98,828	0.429	*ns*	87,916	0.500	**** ****
Pastures	0	1.0		106,190	0.346	
Results of Statistical Tests	Q = 8.75 $p = 0.016$	Q = 8.75 $p = 0.016$		Q = 94.5 $p < 0.0001$	Q = 94.5 $p < 0.001$	

Supplementary Table 4:

Results of the acoustic distance (and scaled similarity values) comparisons across all frequency bands between every site type and the two baseline ecosystems – Reference Forests and Pastures. Distance and similarity values are averaged across all tested bins. Percentages in the final column are calculated as average of the percentage skew towards either baseline in each matching bin. Results of Post-hoc tests between groups are indicated by brackets where *ns* = not significant and **** indicates a p value of < 0.0001.

Supplementary Table 5: Final Results of Acoustic Distance Calculations

Site Type	Distance to Pasture	Similarity to Pasture	Post-Hoc Test	Distance to Reference Forest	Distance to Reference Forest	Post-Hoc Test
Reference Forests	109,193	0.413		0	1.0	
Natural Regeneration (PES)	96,062	0.429	**** ; ****	73,808	0.539	****
Monoculture Plantations (PES)	100,926	0.42	*ns*	92,040	0.489	**** ; ****
Pastures	0	1.0		109,193	0.413	
Results of Statistical Tests	Q = 12.7<br>p = 0.00172	Q = 12.7<br>p = 0.00172		Q = 46.8<br>p < 0.001	Q = 46.8<br>p < 0.001	

Supplementary Table 5:

Results of the acoustic distance (and scaled similarity values) comparisons within the 880 identified time-frequency bins between every site type and the two baseline ecosystems – Reference Forests and Pastures. Distance and similarity values are averaged across all tested bins. Percentages in the final column are calculated as average of the percentage skew towards either baseline in each matching bin. Results of Post-hoc tests between groups are indicated by brackets where *ns* = not significant and **** indicates a p value of < 0.0001.
